# DNA Damage Response After Treatment of Cycling and Quiescent Cord Blood Hematopoietic Stem Cells With Distinct Genotoxic Noxae

**DOI:** 10.1093/stmcls/sxad085

**Published:** 2023-11-14

**Authors:** Fabienne Becker, Meryem Ouzin, Stefanie Liedtke, Katharina Raba, Gesine Kogler

**Affiliations:** Institute for Transplantation Diagnostics and Cell Therapeutics, University Hospital Düsseldorf, Heinrich Heine University Düsseldorf, Germany; Institute for Transplantation Diagnostics and Cell Therapeutics, University Hospital Düsseldorf, Heinrich Heine University Düsseldorf, Germany; Institute for Transplantation Diagnostics and Cell Therapeutics, University Hospital Düsseldorf, Heinrich Heine University Düsseldorf, Germany; Institute for Transplantation Diagnostics and Cell Therapeutics, University Hospital Düsseldorf, Heinrich Heine University Düsseldorf, Germany; Institute for Transplantation Diagnostics and Cell Therapeutics, University Hospital Düsseldorf, Heinrich Heine University Düsseldorf, Germany

**Keywords:** hematopoietic stem cell, cell cycle, DNA damage response, cord blood, quiescence, genotoxins

## Abstract

Hematopoietic stem cells (HSC) from cord blood can be applied as an alternative to bone marrow in transplantation to treat hematological diseases. Umbilical cord blood (UCB) consists of cycling and non-cycling CD34^+^/CD45^low^ cells needed for long-term and short-term engraftment. After sorting and subsequent in vitro culture, quiescent HSCs enter the cell cycle. This enables the analysis of HSCs in 2 different cell cycle stages and the comparison of their responses to different genotoxic noxae. To analyze different mechanisms of DNA damage induction in cells, 2 different genotoxins were compared: etoposide, a topoisomerase II inhibitor that targets mitosis in the S/G_2_-phase of the cell cycle and the alkylating nitrosamine *N*-Nitroso-*N*-methylurea (MNU), which leads to the formation of methyl DNA adducts resulting in DNA double breaks during DNA replication and persistent mutations. Cycling cells recovered after treatment even with higher concentrations of etoposide (1.5µM/ 5µM/10µM), while sorted cells treated with MNU (0.1mM/0.3mM/0.5mM/1mM/3Mm/ 5mM) recovered after treatment with the lower MNU concentrations whereas high MNU concentrations resulted in apoptosis activation. Quiescent cells were not affected by etoposide treatment showing no damage upon entry into the cell cycle. Treatment with MNU, similarly to the cycling cells, resulted in a dose-dependent cell death. In conclusion, we found that depending on the genotoxic trigger and the cycling status, CD34^+^cells have distinct responses to DNA damage. Cycling cells employ both DDR and apoptosis mechanisms to prevent damage accumulation. Quiescent cells predominantly undergo apoptosis upon damage, but their cell cycle status protects them from certain genotoxic insults.

## Graphical Abstract

**Graphical Abstract:**
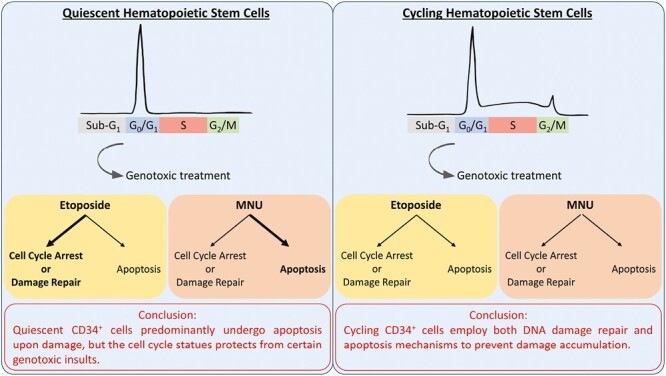

**Significance Statement**

The neonatal HSCs isolated from cord blood are predominantly quiescent but their introduction into the cell cycle enables the comparison between quiescent and cycling cells, which differ in their response mechanisms to DNA damage, eg, replication stress/double-strand breaks. The differentiation between these populations is important during the toxicological assessment of therapeutical treatments in terms of hematotoxicity for a better prediction of genotoxic exposure but also to predict the contribution to carcinogenesis caused by nitrosamines, whose presence in food and pharmaceuticals is prohibited by the European Food Safety Authority (EFSA) and the European Medicines Agency (EMA).

## Introduction

Every cell is constantly exposed to endogenous stress such as replication leading to daily DNA damage accumulation.^1^ Additionally, exogenous triggers, such as genotoxic substances taken up through food, drug and environmental contaminants or during chemotherapy, can lead to major DNA damage.

UCB HSCs can be applied as an alternative source to bone marrow HSCs for stem cell transplantation. Although these cells have lower short-term engraftment capacity compared to peripheral blood stem cells or bone marrow, they have many advantages, eg, less-invasive collection procedure, low graft-versus-host disease (GvHD), and rapid availability.^2^

HSCs isolated from UCB are mainly maintained in a quiescent state to protect against exogenous and endogenous insults and avoid leukemic transformation and stem cell pool depletion.^3^

Moreover, mutations induced during chemotherapy are considered as the most severe side effects leading to myelosuppression and therapy-related AML (t-AML). The activation of the DNA damage repair (DDR) mechanisms results in one of 2 outcomes: damage repair with following genomic restoration and survival or damage persistence and following induction of apoptosis, senescence or cell cycle arrest thus preventing damage accumulation.^4^

Beside quiescence, HSCs are assumed to have additional evolutionary characteristics enabling protection against damage and resistance to acute injury. This is related to expression of different DDR genes and epigenetic factors. Milyavsky *et al.* reported a delayed DNA double-strand break (DSB) repair and persistent γH2AX-foci combined with an increased p53-dependent apoptosis upon γ-irradiation in HSCs compared to their progenitors allowing the in vivo repopulating HSC function.^5^ Any DSB occurring during the quiescent cell stage is repaired via the non-homologous end-joining (NHEJ) pathway, which as an error-prone repair pathway, may be the cause for accumulated DNA damage, while DDR shifts toward homologous recombination (HR) upon cell cycle entry.^6,7^

In vitro, HSCs can be isolated from CB via enrichment/sorting of CD34^+^ cells in their quiescent state.^8^ The maintenance of HSCs and their progeny in vitro enables the investigation of DDR and the underlying intracellular mechanisms to various genotoxins.^9^

Etoposide, a cytostatic drug approved for treatment of various neoplasms, acts through the inhibition of topoisomerase II, resulting in DSB accumulation as the DNA unwinding, cleavage and re-ligation for the resolution of the topological tension is blocked.^10-14^ The outcome varies from DSB repair to cell death depending on the severity of damage and needs to be considered as a key determinant of hematological toxicity of cytotoxic drugs.^15^

Nitrosamines are considered as a broad-acting group of carcinogens. Nitrosamine exposure occurs through diet, eg, cured meats and beer and are formed by the reaction of secondary amines, amides, and carbamates with the nitrogen in amino acids.^16^ Moreover, increased awareness to nitrosamine impurities during the manufacturing process of several drugs, solvents, and catalysts was reported in 2018. This resulted in the establishment of a nitrosamine task force by the U.S. Food and Drug Administration (FDA) for guidance publication and development of testing methods for nitrosamine impurities. The EFSA provided a scientific opinion on the request of the European Commission to evaluate human health risks related to nitrosamine food contaminants.^17^

*N*-Methyl-*N*-nitrosourea (MNU) is an alkylating nitrosamine, which exerts its mutagenic effects by methylation of nucleotides, resulting in destabilization/breakage of DNA.^18^ Without removal by the repair enzyme O^6^-methylguanine-DNA methyl-transferase (MGMT), the modified base persists in the DNA and results in a point mutation, but could also induce epigenetic changes if a motif is lost.^19,20^ Besides this risk, the presence of O^6^-methylguanine might cause DSBs during replication.^21^

Although several DDR pathways have been identified for various cell types in vitro, it is important to analyze in detail how HSCs react to exogenous/endogenous insults with respect to their cell cycle status.^22^

DDR of both quiescent and cycling HSCs from cord blood was investigated. We hereby focused on 2 distinct genotoxins interfering in different cell cycle phases leading to DSBs.

## Materials and Methods

### Isolation of Hematopoietic Stem Cells

CB was collected from the umbilical cord with informed consent from the mother and donated to the José Carreras Cord Blood Bank Düsseldorf (Approval by the ethic commission 2975).

HSCs were isolated from CB by pre-enrichment of mononuclear cells. Density gradient centrifugation was performed using 1.077 g/cm^3^ Histopaque-1077 Hybri-Max (Sigma-Aldrich). Cells were washed with PBS/EDTA (Miltenyi) and CD34^+^cell fraction was isolated via magnetic-activated cell sorting using human CD34 MicroBead Kit (Miltenyi). Isolation was performed according to the manufacturer protocol.

Prior to MNU treatment, HSCs were subjected to fluorescence-activated cell sorting (FACS) to increase cell purity using the Moflo XDP (Beckmann Coulter). The instrument was used with standard optical configuration and samples were sorted using a 100-µm nozzle. First, live cells were selected based on FSC/SSC. Doublets were excluded by the SSC-width parameter and dead cells by staining with 7-aminoactinomycin D (Beckman Coulter). Phycoerythrin (PE)-conjugated anti-CD34 antibodies (BD Bioscience) and fluorescein-isothiocyanate (FITC)-conjugated anti-CD45 antibodies (BD Bioscience) were used to determine HSCs. The HSC population was sorted via CD34^+^CD45^low^ (Supplementary Fig. S1).

Flow cytometry for CD34/CD45-markers was performed using a CytoFlex Flow Cytometer (Beckman Coulter) following the protocol according to the International Society of Hematotherapy and Graft Engineering (ISHAGE).^23^

### Expansion of Hematopoietic Stem Cells

Cells were expanded on 24-well plates (Greiner AG) in CellGenix-GMP-SCGM medium (CellGenix) with 100 µg/mL penicillin (Lonza Group), 100 µg/mL streptomycin (Lonza Group), and 25 ng/mL of the following cytokines (Miltenyi): human stem cell factor (SCF), human Flt3-ligand (Flt3-L), human thrombopoietin (TPO), human interleukin 6 (IL-6), and human interleukin 3 (IL-3) and incubated at 37 °C in a humidified atmosphere with 5% CO_2_.

### Genotoxic Treatment of Cells

Quiescent CD34^+^cells were subjected to treatment immediately after isolation, whereas cycling cells were expanded for 5 days prior to treatment.

One hundred millimolars and 1M stock solutions of Etoposide (TCI EUROPE NV) and MNU (MedChemExpress) were prepared in DMSO (Wak-Chemie Medical GmbH), respectively. Etoposide treatment was conducted for 24 h with following concentrations: 1.5 µM/5 µM/10 µM. MNU treatment was conducted for 1 h with following concentrations for qPCR: 1 mM/3 mM/5 mM and 0.1 mM/0.3 mM/0.5 mM for all other experiments. After treatment, cells were washed with PBS (Miltenyi) prior to further cultivation or subsequent analysis (Supplementary Fig. S2).

### Coculture of Hematopoietic Stem Cells With Bone Marrow Mesenchymal Stromal Cells (BM-MSC Feeder)

One day before co-cultivation with CD34^+^cells, BM-MSCs were plated on a 24-well plate in DMEM medium containing 30% FCS (Gibco GmbH), 100 µg/mL Penicillin (Lonza Group), and 100 µg/mL Streptomycin (Lonza Group) at a density of 1 × 10^5^ cells/well. 2 × 10^5^ cells/well CD34^+^ cells were seeded on BM feeder and treated either with the feeder or before plating the cells on the feeder.

### Cell Count and Assessment of Proliferation

Cell counting was performed using 10 µL trypan blue dye (Sigma-Aldrich), with 10 µL of cell suspension at different culture timepoints, and an improved Neubauer counting chamber (NanoEnTek). The cumulative population doublings (CPD) were performed applying the formula: PD = [log(n1/n0)]/log2; CPD = ΣPD; n1: number of counted cells. n0: number of plated cells.

### Cell Fate Analysis

The subpopulations present in the CD34^+^fraction were analyzed by flow cytometry using following monoclonal antibodies: CD34-PE (Miltenyi), CD38-APC (BD Pharmingen), CD10-PE-Cy™7 (BD Pharmingen), CD45Ra-APC-A750 (Beckman Coulter), CD90-FITC (Miltenyi), Propidium Iodide (BioLegend). Cells were co-stained with all antibodies for 15min and washed with PBS. For flow cytrometry, CytoFlex Flow Cytometer (Beckman Coulter) was used. The gating strategy is shown in Supplementary Fig. S3.

### Cell Cycle Analysis

For cell cycle analysis, propidium iodide (PI) was applied for nuclear staining as described by C. Riccardi and I. Nicoletti [43]. 2.5 × 10^4^ cells were washed twice with PBS (Miltenyi) (7 minutes/4 °C/550 g). Cells were resuspended in 25 μL staining solution and incubated in the dark for 1 h/4 °C prior to flow cytometry (Beckman Coulter).

### Apoptosis Assay

Apoptosis detection/quantification was performed using Annexin V-FITC Apoptosis Detection Kit (BD Bioscience Pharmingen) according to the manufacturer protocol. Flow cytometry analysis was performed using CytoFlex Flow Cytometer (Beckman Coulter).

### Total RNA Isolation and Reverse Transcription

At different timepoints during/after genotoxic treatment, cells from each condition were collected for total RNA isolation. RNeasy-Kit (Qiagen) was used according to the manufacturer instructions including the optional 15 minutes DNase digest. Determination of RNA concentrations and purity was carried out using a Nanodrop device (NanoDropTechnologies).

Reverse transcription was applied using the first-strand cDNA synthesis kit (Invitrogen) and oligo(dT) 20 primer (Thermo Fisher Scientific) following the manufacturer instructions. One microgram of total RNA was converted into first-strand cDNA in a 20-µL reaction.

### Quantitative PCR Analysis

qPCR analysis was carried out with intron-spanning primers specific for each gene (Thermo Fisher Scientific). The respective primer sequences are given in Supplmentary Table S1. RPL13a was used as a reference gene.

Fifty nanograms of cDNA were applied for RT-PCR in a total volume of 25 µL containing Sybr Green PCR Mastermix (Thermo Fisher Scientific), 0.2 µM of primer (forward + reversed), distilled water, and 50 ng template (10 minutes/95 °C-15 s/95 °C-1 minutes/60 °C for 40 cycles).

To analyze the comparative CT experiments, Step One Software v.1.5.1 was used. Relative changes in gene expression were calculated by applying the comparative ∆∆CT method.^24^ Differential gene expression was calculated by the formula 2^−∆∆CT^ normalized to untreated cells. Fold changes < 1 were transformed by the formula −1/2^−∆∆CT^ in the case of downregulated genes and plotted together with positive fold changes and upregulated genes, respectively.

### Immunocytochemistry

5 × 10^4^ CD34^+^cells were harvested at different timepoints (0.5-48 h) during treatment with MNU or etoposide. For the latter, a media change was performed 24 h after its addition by washing with PBS (550 g/4 °C/7 minutes). After resuspension in PBS/0.5% HSA, cells were transferred into a funnel (Thermo Fisher Scientific) of Cytospin3 (Thermo Shandon) inlets assembled with a microscope SuperFrost slide (Thermo Fisher Scientific) and a filter card (Epredia). Cells were transferred onto the glass slide (600g/5min/room temperature (RT)). A 8mm cloning ring (Merck) was placed on the slide and cells were fixed with 4% formaldehyde-solution (AppliChem) for 15min, washed thrice with PBS for 5min, permeabilized with ice-cold methanol for 20min/-2 0°C and washed with PBS for 5 minutes. Non-specific antigen were incubation in blocking buffer (5%(v/v) NGS (Thermo Fisher Scientific) in PBS/0.3%(v/v) triton X-100 (Thermo Fisher Scientific)) for 1h. Primary antibody co-staining with anti-γH2AX (Ser139) (Merck) and anti-53BP1 rabbit polyclonal antibody (Cell Signaling Technology) was performed with 0.5µg/mL each in blocking buffer overnight at 4 °C. Afterward, cells were washed twice with PBS for 5min, once with PBS/0.4M NaCL for 2min, and once with PBST (PBS/0.3%(v/v) triton X-100) for 5min, before secondary antibody incubation with 0.5µg/mL of Alexa Fluor 488 AffiniPure Goat Anti-rabbit IgG (JacksonImmunoResearch) and Rhodamine Red™-X (RRX) AffiniPure Goat Anti-Mouse IgG (JacksonImmunoResearch) each in blocking buffer for 1h at RT in the dark. After washing thrice with PBS for 5min and rinsing twice with PBST, cloning rings were removed, Fluoromount-G™ mounting medium (Thermo Fisher Scientific) applied and slides covered with a coverslip. Cells were examined using Axio Observer 7 (Zeiss), and foci analysis was performed using (Fiji Is Just) ImageJ (Version 2.9.0).

## Results

### Characteristic Differences Between Quiescent and Cycling CD34^+^ Cells

**Figure 1.**
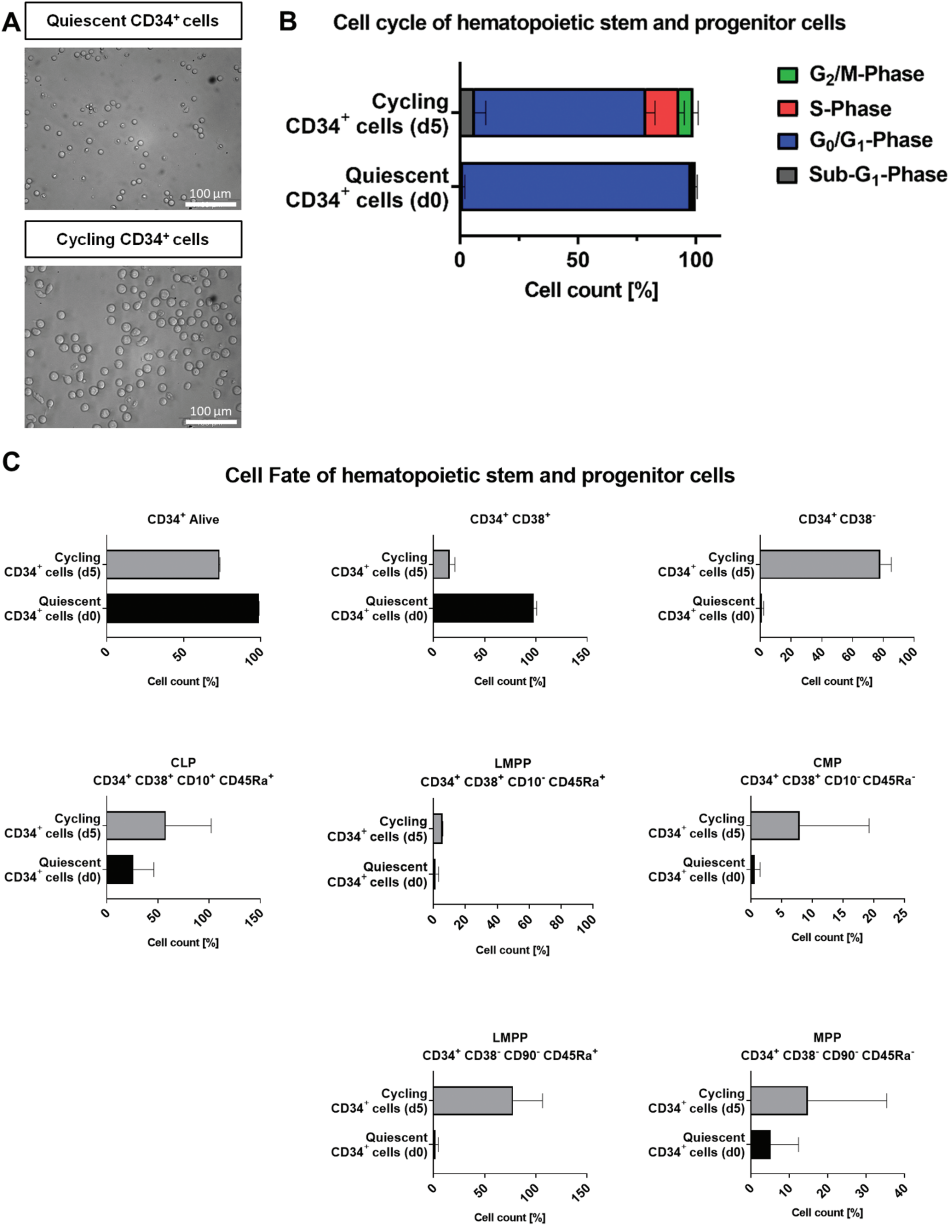
Characterization of quiescent and cycling hematopoietic stem and progenitor cell populations. (**A**) Representative morphology of untreated quiescent and cycling CD34^+^ cells. To ensure examination of quiescent CD34^+^ cells, they were immediately analyzed after isolation from cord blood; cycling cells were analyzed after 5 days of culture; scale bar = 100 μm. (**B**) Comparison of cell cycle analysis of untreated quiescent and cycling CD34^+^ cells. (**C**) Comparison of cell surface markers of quiescent and cycling CD34^+^ cells. Abbreviations: CLP, common lymphoid progenitor; CMP, common myeloid progenitor; LMPP, lymphomyeloid-primed progenitor; MPP, multipotent progenitor.

Isolation of cells from CB via CD34 marker yields a heterogeneous cell population. To distinguish these subpopulations, a set of different markers was applied. Freshly isolated quiescent CD34^+^cells have a spherical morphology (Fig. 1A) and consist of 97.7% CD34^+^CD38^+^ (25.92% CLP, 1.4% LMPP, 0.65% CMP) and 0.91% CD34^+^CD38^−^ cells (2% LMPP, 5.14% MPP) (Fig. 1C). They are considerably smaller than their cycling counterpart and consist of 16.08% CD34^+^CD38^+^ (57.75% CLP, 5.7% LMPP, 7.97% CMP) and 77.91% CD34^+^CD38^-^ cells (77.65% LMPP, 14.83% MPP; Fig. 1C).

Directly after isolation, quiescent CD34^+^cells were primarily in the G_0_/G_1_-phase of the cell cycle (≥95%). Expansion with broad-acting cytokines induced cell cycle entry, where the majority of cells (72.55%) were still in the G_0_/G_1_-phase. However, there were also actively dividing cells in S-phase (14.15%) and G_2_/M-phase (5.99%) and a smaller fraction of apoptotic cells (6.23%), represented by the sub-G_1_-phase (Fig. 1B).

### Dose-Dependent Damage of Cycling CD34^+^ Cells

#### Etoposide

**Figure 2.**
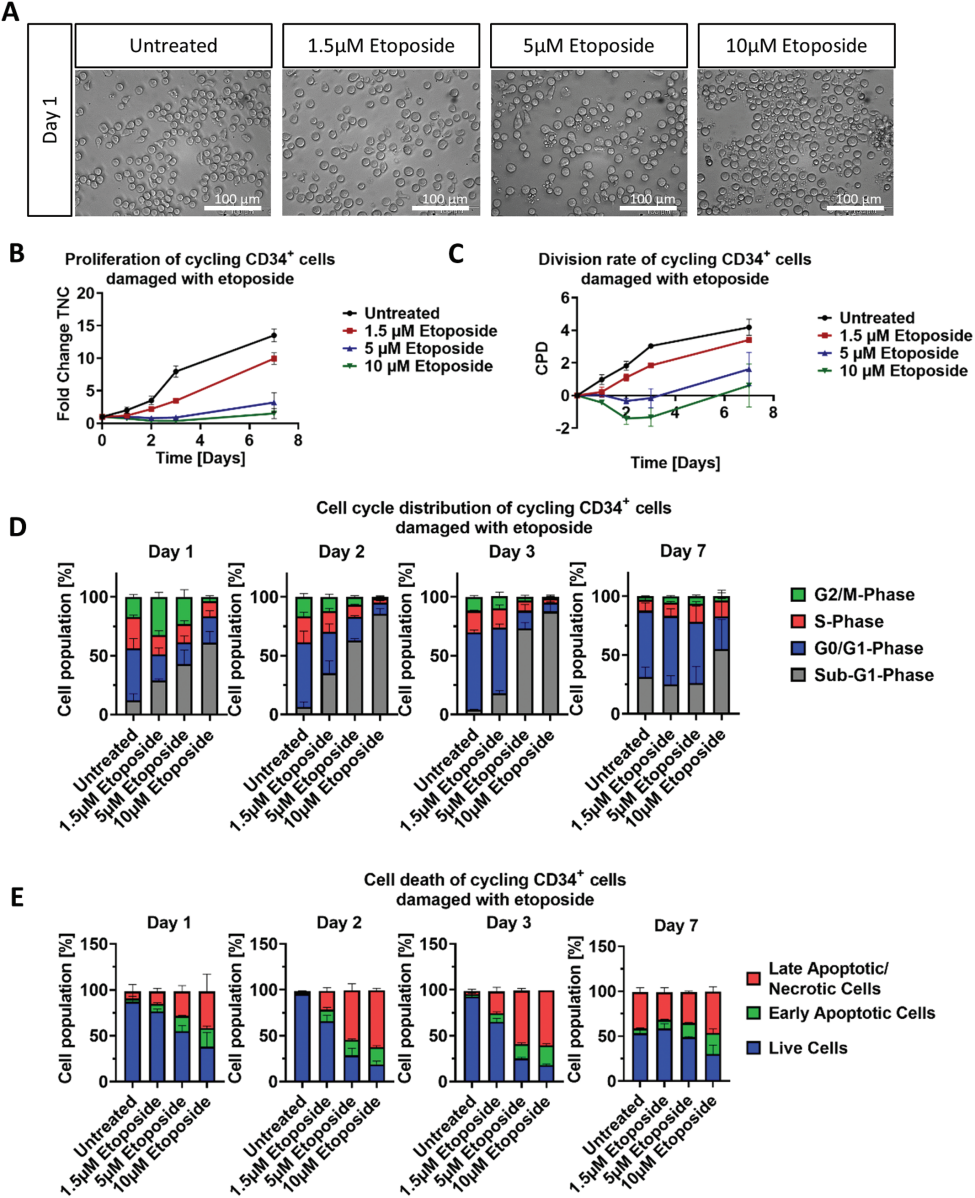
Effect of etoposide treatment on morphology, growth and cell cycle of cycling CD34^+^ cells. (**A**) Representative morphological images of untreated and with 1.5, 5, and 10 μM etoposide treated cycling CD34 + cells. Analysis was performed after 24 h treatment. Scale bars = 100 μm. (**B**, **C**) Representative growth kinetics depicted via the total fold change over time and the CPD. After treatment of cycling cells with different etoposide concentrations, the growth curves revealed a dose-dependent effect on cycling CD34^+^ cells. (**D**) Cell cycle analysis of treated cycling CD34^+^ cells via staining with PI and analysis by flow cytometry. (B-D) Representative data from 3 independent experiments with different CB donations. Abbreviations: CB, cord blood; CPD, cumulative population doubling, PI, propidium iodide.

Morphological analysis of cycling HSC, after treatment with various concentrations of etoposide (1.5 µM/5 µM/10 µM) for 24 h, revealed visible cell damage with different stages of apoptotic morphology, ie, membrane blebbing and formation of apoptotic membrane protrusions, in all conditions (Fig. 2A). Within 3 days, increasing signs of apoptosis were observed in a dose-dependent manner, ie, the cells treated with higher concentrations showed severe apoptotic blebbing/vesicles (Supplementary Fig. S4). Furthermore, on day 7, CD34^+^cells treated with 1.5 µM reached a fold change of 9.9, whereas 5 and 10 µM treatment resulted in a fold change of only 3.2 and 1.5, respectively, compared to the fold change of untreated cells (13.5) (Fig. 2B).

To investigate the origin of reduced cell count, both cell cycle analysis and apoptosis assay was used. The distribution of cells in each cell cycle phase did not fluctuate in the untreated control (on average 7.7% in the sub-G_1_-phase, 54.8% in the G_0_/G_1_-phase, 22.4% in the S-phase, and 15.0% in the G_2_/M-phase) (Fig. 2D). In comparison, cells treated with 1.5 µM etoposide showed a significant increase of cells in the G_2_/M-phase (32.1%) and a decrease of cells in both G_0_/G_1_-phase (22.2%) and S-phase (16.5%) on day 1. On the following days, the distribution of cells in each cell cycle was comparable to that of untreated cells. Cells treated with 5 µM etoposide showed a similar trend of cell distribution in the cell cycle on d1 (18.3% in the G_0_/G_1_-phase, 15.6% in the S-phase, and 22.9% in the G_2_/M-phase), except for a significant increase in cells in the sub-G_1_ phase (43.1%). In the following 2 days, the amount of cells in sub-G_1_-phase significantly increased (73.3%), whereas the overall cell numbers decreased. After treatment with 10 µM etoposide, most cells were in the sub-G_1_-phase (61.2%) and only a small fraction was actively involved in the other cell cycle phases (22.1% in the G_0_/G_1_-phase, 13.1% in the S-phase, and only 3.4% in the G_2_/M-phase) starting from day 1.

Dose-dependent increase of cells in the sub-G_1_ phase was further characterized as cells undergoing apoptosis via Annexin V/PI staining, showing that over the 7 days, the number of cells in the late apoptotic stage increased with growing etoposide concentration, with more than 50% of cells undergoing apoptosis after exposure to 5 and 10 µM etoposide (Fig. 2E).

To study the effect of the microenvironment on damaged CD34^+^ cells, a co-cultivation experiment with BM-feeder was performed, with treatment of cells while they are in culture with the feeder. Analyzes revealed no significant contrast to the effect on cycling CD34^+^cells with the feeder present as compared to cells treated without the feeder (Supplementary Figs. S9 and S8).

#### MNU

**Figure 3.**
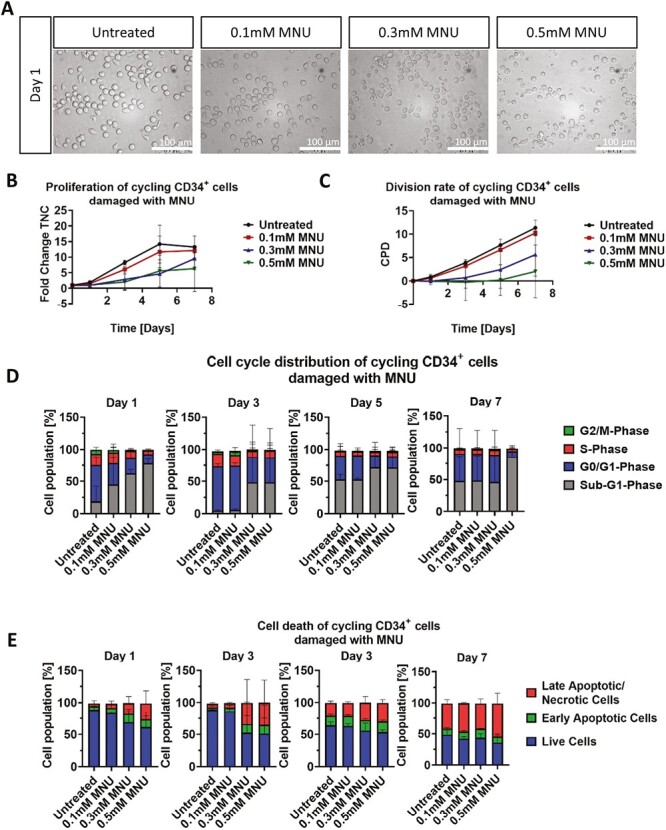
Effect of MNU treatment on morphology, growth and cell cycle of cycling CD34^+^ cells. (**A**) Representative morphological images of untreated and with 0.1, 0.3, and 0.5 mM MNU treated cycling CD34 + cells. Analysis was performed ~24 h after the 1 h treatment. Scale bars = 100 μm. (**B**, **C**) Representative growth kinetics depicted via the total fold change over time and the CPD. After treatment of cycling cells with different MNU concentrations, the growth curves revealed a dose-dependent effect on cycling CD34^+^ cells. (**D**) Cell cycle analysis of treated cycling CD34^+^ cells via staining with PI and analysis by flow cytometry. (B-D) Shown are representative data from 3 independent experiments with different CB donations. Abbreviations: CB, cord blood; CPD, cumulative population doubling; MNU, *N*-methyl-*N*-nitrosurea; PI, propidium iodide.

Morphological analysis of cycling CD34^+^ cells after treatment with various concentrations of MNU (0.1 mM/0.3 mM/0.5 mM) for 1 h, revealed no visible cell damage on day 1 after treatment (Fig. 3A). At day 3, an extensive damage was visible, with different stages of apoptotic morphology observed in a dose-dependent manner, ie, cells treated with higher concentrations showed severe apoptotic-blebbing (Supplementary Fig. S7). Cell counts revealed a fold change of 18.5 in the untreated control, whereas treatment with 0.1 mM MNU resulted in a lower fold change of 15.3, and cells treated with both 0.3 and 0.5 mM MNU resulted in a near complete proliferation stop (Fig. 3B).

Cell cycle analysis showed that on day 1 both 0.3 and 0.5 mM treatment resulted in increased cell numbers in the sub-G_1_-phase (62.45% and 78.24%, respectively). At day 7, cells treated with 0.3 mM were able to reconstitute their cell cycle, while most cells treated with 0.5 mM remained in the sub-G_1_ phase (86.21%). In contrast, treatment with 0.1 mM showed no significant distinction in cell cycle distribution compared to the untreated control (Fig. 3D). Accordingly, apoptosis assay showed increased late apoptotic populations after treatment with 0.3 and 0.5 mM MNU until day 3. Between days 5 and 7, levels of apoptotic populations in all conditions were comparable to the untreated control (Fig. 3E).

Co-cultivation of cycling CD34^+^ with a BM-feeder was performed with 2 different treatment methods. After treating both CD34^+^ cells and the feeder, CD34^+^ cells showed no improved recovery after damage compared to cultivation without a feeder (Supplementary Figs S10 and S11). In contrast, transferring treated CD34^+^ cells onto a healthy feeder documented the protective capacity of the microenvironment on damaged cells, as all concentrations depicted the same morphology/cell count/cell cycle distribution/cell death tendencies as untreated cells (Supplementary Figs. S12 and S13).

### Dose-Dependent Damage of Quiescent CD34^+^ Cells

#### Etoposide

**Figure 4.**
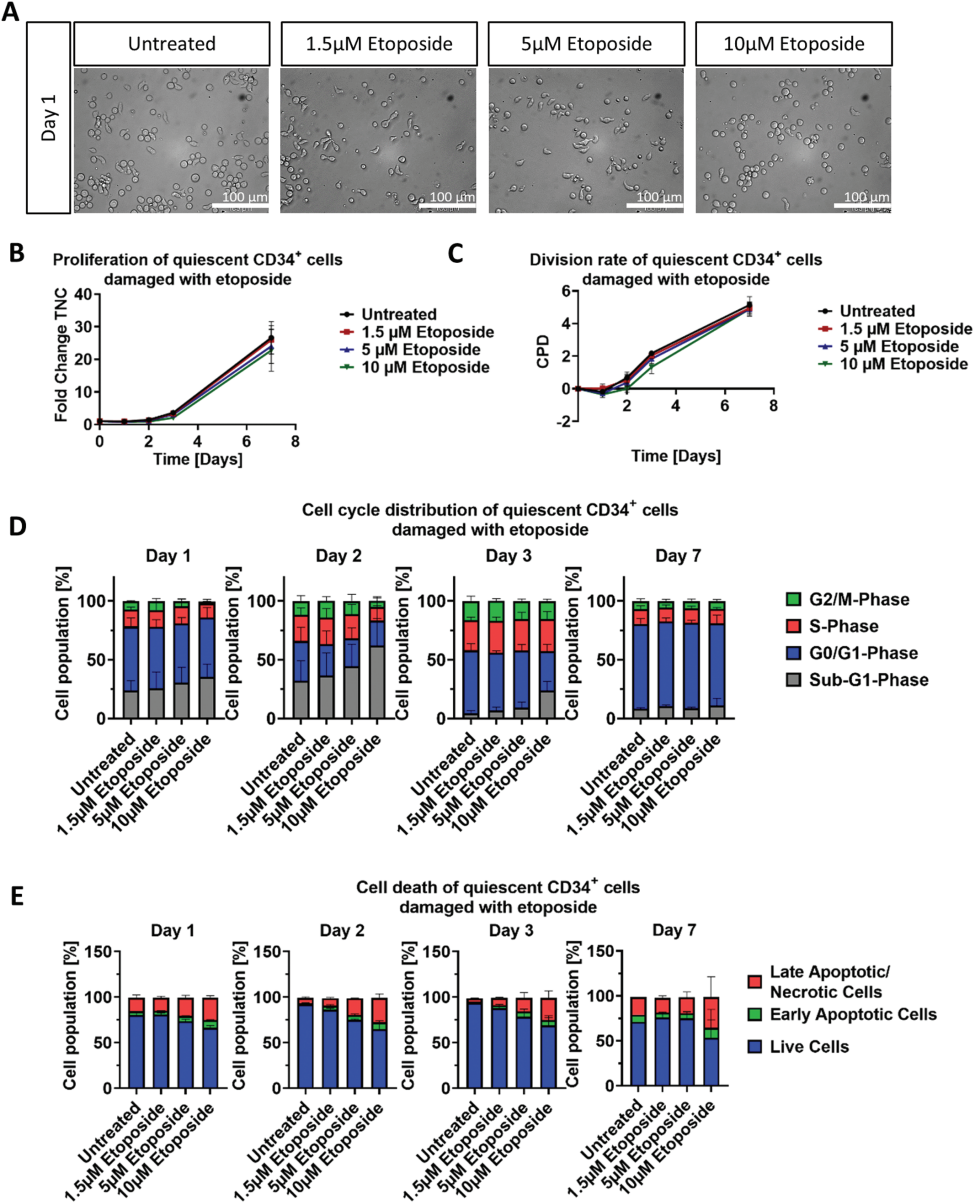
Effect of etoposide treatment on morphology, growth, and cell cycle of quiescent CD34^+^ cells. (**A**) Representative morphological images of untreated and with 1.5, 5, and 10 μM etoposide treated quiescent CD34^+^ cells. Analysis was performed after a 24 h treatment. Scale bars = 100 μm. (**B**, **C**) Representative growth kinetics depicted via the total fold change over time and the CPD. After treatment of quiescent cells with different etoposide concentrations, the growth curves revealed no significant effect on quiescent CD34^+^ cells. (**D**) Cell cycle analysis of treated quiescent CD34^+^ cells via staining with PI and analysis by flow cytometry. (B-D) Shown are representative data from 3 independent experiments with different CB donations. Abbreviations: CB, cord blood; CPD, cumulative population doubling, PI, propidium iodide.

Morphological analysis of quiescent HSC, after treatment with various concentrations of etoposide (1.5, 5, or 10 µM) for 24 h, revealed some smaller cells and few cells with membrane blebbing only in the fraction treated with 10 µM etoposide (Fig. 4A). Three days after treatment, no further signs of apoptosis were visible (Supplementary Fig. S5). Cell counts showed that quiescent CD34^+^ cells were not significantly damaged in their proliferative capacity, as fold changes at day 7 were comparable between all conditions (26.7 for untreated, 26.0 for 1.5 µM, 24.0 for 5 µM, 22.7 for 10 µM). However, proliferation start was delayed in a dose-dependent manner up to 2 days after culture start (Fig. 4B, C).

Cell cycle analysis revealed that only treatment with 1.5 µM resulted in comparable distribution of cells in each cell cycle to those of untreated cells (on average 13.6% in the subG_1_-phase, 54.3% in the G_0_/G_1_-phase, 18.5% in the S-phase, and 10.5% in the G_2_/M-phase). In contrast, 5 µM treatment resulted in a slight shift of cell distribution (34% in the sub-G_1_-phase, 28.0% in the G_0_/G_1_-phase, 25.1% in the S-phase, and 12.8% in the G_2_/M-phase), and treatment with 10 µM had a more significant dispersion, with an increase of cells in the sub-G_1_-phase (49.7%), and a decrease in the G_0_/G_1_-phase(28.0%), the S-phase (15.9%), and the G_2_/M-phase (6.4%). Both 5 and 10 µM etoposide treated cells regained a comparable distribution of cells in each cell cycle to that of untreated cells by day 7 (Fig. 4D). This slight shift of more sub-G_1_ cells in a dose-dependent manner was further confirmed via apoptosis assay, showing increased levels of late apoptotic cells for 5 and 10 µM treatment. Nevertheless, as seen in cell cycle analysis, the apoptotic population levels in all conditions became comparable to those of untreated cells at day 7 (Fig. 4E).

#### MNU

**Figure 5.**
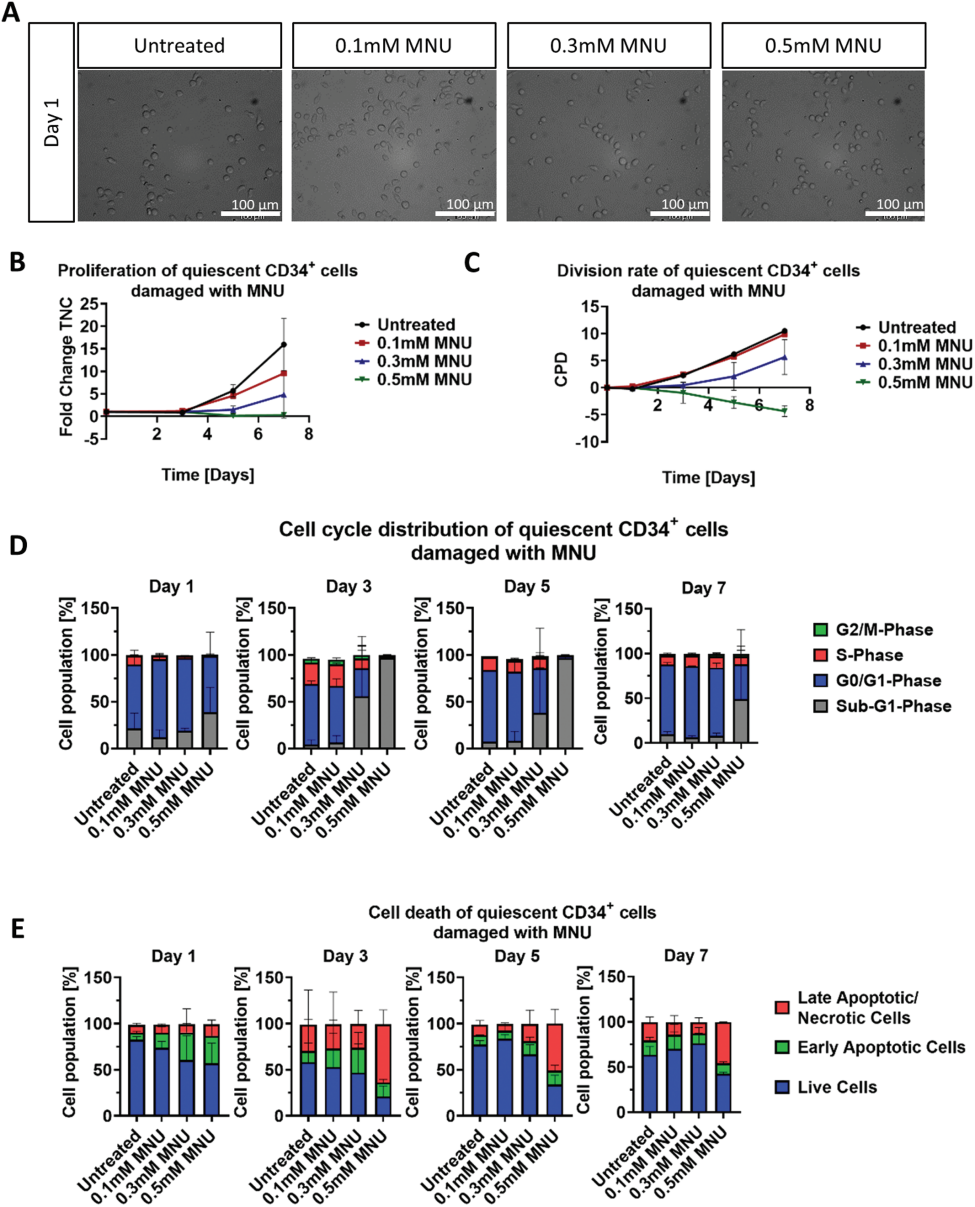
Effect of MNU treatment on morphology, growth and cell cycle of quiescent CD34^+^ cells. (**A**) Representative morphological images of untreated and with 0.1, 0.3, and 0.5 mM MNU treated quiescent CD34^+^ cells. Analysis was performed 24 h after the 1 h treatment. Scale bars = 100 μm. (**B**, **C**) Representative growth kinetics depicted via the total fold change over time and the CPD. After treatment of quiescent cells with different MNU concentrations, the growth curves revealed a dose-dependent effect on quiescent CD34^+^ cells. (**D**) Cell cycle analysis of treated quiescent CD34^+^ cells via staining with PI and analysis by flow cytometry. (B-D) Shown are representative data from 3 independent experiments with different CB donations. Abbreviations: CB, cord blood; CPD, cumulative population doubling; MNU, *N*-methyl-*N*-nitrosurea; PI, propidium iodide.

Morphological analysis of quiescent CD34^+^cells, after treatment with various concentrations of MNU (0.1, 0.3, or 0.5 mM) for 1 h, revealed no visible cell damage upon treatment termination (Fig. 5A). Only after day 3, the extensive damage was visible, with different stages of apoptotic morphology observed in cells treated with 0.5 mM MNU ie, higher concentrations showed severe apoptotic blebbing and vesicles (Fig. 5A). Cell counts for quiescent CD34^+^cells treated with MNU revealed an overall fold change of 19.44 for the untreated control, treatment with 0.1 and 0.3 mM had a slightly lower fold change of 17.78 and 12.42, respectively, whereas 0.5 mM treated cells only reached a fold change of 0.33 at day 7 (Fig. 5B).

Although cell counts did not reveal any major discrepancy between untreated cells and 0.1/0.3 mM, cell cycle analysis revealed a severe cell cycle arrest in the G_0_/G_1_-phase (83.51% for 0.1 mM, 77.71% for 0.3 mM, 60.42% for 0.5 mM) at day 1 (Fig. 5C). However, this arrest was resolved for 0.1 mM/0.3 mM, whereas with 0.5 mM nearly all cells converted from cell cycle arrest into sub-G_1_ phase (>95%), only a small fraction recovered at day 7.

Accordingly, apoptosis assay showed increased early and late apoptotic populations in a dose-dependent manner, with 0.5 mM treatment resulting in the highest number of apoptotic cells after cell cycle arrest (45.42%) at day 7 (Fig. 5E).

### Treatment of Hematopoietic Stem and Progenitor Cells With Genotoxins Induces the Formation of γH2AX and 53BP1 Foci

To investigate DSB occurence after etoposide (1.5, 5, 10 µM) or MNU (0.1, 0.3, 0.5 mM) treatment, quiescent and cycling CD34^+^ cells were co-stained for γH2AX and 53BP1. Generally, 4 distinguished foci types were observed, (A) cells displaying only γH2AX-foci, (B) partial or complete co-localization of both γH2AX- and 53BP1-foci, and (C) rare instances with cells displaying only 53BP1-foci that resembled apoptotic rings (Supplementary Fig. S15). From the counted mean number of foci/nucleus, a threshold of ≥ 5 was selected as significant to determine the amount of foci positive cells. From this, co-localization of γH2AX/53BP1 was calculated. Complete co-localization was defined as ≥ 80% matching γH2AX/53BP1-foci, whereas partial co-localization was defined as < 80% γH2AX/53BP1-foci.

**Figure 6.**
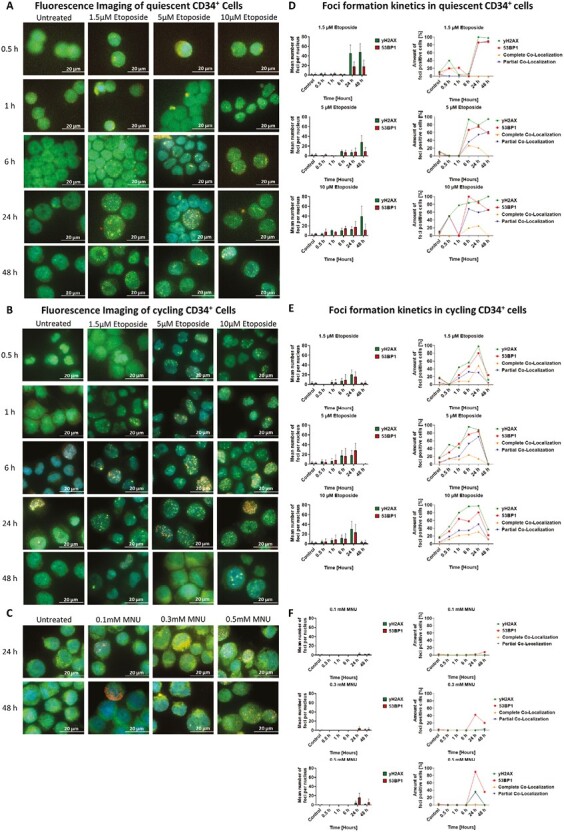
γH2AX and 53BP1 foci formation kinetics in CD34^+^ cells after treatment with genotoxic noxae etoposide or MNU. Quiescent or cycling CD34^+^ cells were treated with etoposide (1.5, 5, and 10 μM) or MNU (0.1, 0.3, and 0.5 mM), and harvested between 0.5 and 48 h and co-stained for γH2AX (green) and 53BP1 (red), using HOECHST (blue) to visualize the nucleus. Representative fluorescence co-staining images of (**A**) quiescent CD34^+^ cells treated with different doses of etoposide, (**B**) cycling CD34^+^ cells treated with different doses of etoposide, and selective images of (**C**) cycling CD34^+^ cells treated with different doses of MNU. Time course for the formation of different foci in (**D**) quiescent CD34^+^ cells treated with different doses of etoposide, (**E**) cycling CD34^+^ cells treated with different doses of etoposide, and (**F**) cycling CD34^+^ cells treated with different doses of MNU; the mean number of foci per nucleus includes at least 25 counted cells, from which the amount of foci positive cells (%) were determined. To determine positive stained cells, for either γH2AX or 53BP1, a threshold of ≥ 5 foci per nucleus was applied. From these, the co-localization of the 2 foci was calculated using a ≥ 80% overlap threshold of γH2AX and 53BP1 foci in one cell for the complete co-localization, and a < 80% overlap threshold of γH2AX and 53BP1 foci in one cell for the partial co-localization. Scale bars = 20 μm. Abbreviations: MNU, *N*-methyl-*N*-nitrosurea.

Analyzing the response of quiescent CD34^+^ cells to treatment with etoposide revealed a time- and dose-dependent foci formation (Fig. 6A, D). In cells treated with 1.5 µM, most foci were recorded 24 h after genotoxin addition, with more γH2AX-foci (45.4 ± 17.2) than 53BP1-foci (17.6 ± 10.2), which resulted in only partial co-localization. Treatment with 5 and 10 µM resulted in earlier foci detection, 6 and 1 h after addition of etoposide, respectively, with both conditions displaying highest numbers of foci/nucleus after 48 h. Furthermore, there were more γH2AX-foci (28.1 ± 13.7 for 5 µM, 39.6 ± 20.8 for 10 µM) than 53BP1-foci (9.5 ± 7.6 for 5 µM, 11.9 ± 11.8 for 10 µM) detected, with the majority only partially co-localized (~60%). On the other hand, cycling CD34^+^ cells treated with etoposide, displayed a similar foci formation time course for all concentrations, with foci detected between 0.5 and 1 h after addition of genotoxin and highest numbers of foci/nucleus at 24 h, after which only small numbers or no foci were counted (Fig. 6B, E). The overall number of foci was reduced compared to those counted in quiescent cells (γH2AX-foci/nucleus: 20.3 ± 9.5 for 1.5 µM, 18.5 ± 9.7 for 5 µM, 30.5 ± 15.8 for 10 µM). The difference to 53BP1-foci/nucleus was not as high (53BP1-foci/nucleus: 16.1 ± 9.7 for 1.5 µM, 27.9 ± 14.9 for 5 µM, 23.1 ± 16.8 for 10 µM) with both foci being completely and partially co-localized in cycling CD34^+^ cells. In contrast to the foci formation upon etoposide treatment, MNU treatment revealed no foci formation in quiescent cells and only low foci numbers in cycling cells with the majority being 53BP1-foci rather than γH2AX-foci (Fig. 6C, F and Supplementary Fig. S14).

## Discussion

This work focuses on the characterization of CD34^+^ cell response to damage induced by 2 different genotoxic noxae, with special interest in deciphering the importance of cell cycle status in cell fate decision.^22^

Although the CD34^+^ cell population has been characterized to a certain degree in past works, differences in specific CD marker expression were found in this work.^25,26^ CD38 expression was greatly upregulated in the tested CD34^+^ cells directly after isolation, with > 95% being CD34^+^CD38^+^. This would lead to the assumption that most cells isolated from UCB were already at a later differentiation state with no short-term (ST-HSC) or multipotent progenitors (MPPs) present. Contradictory to this, the CD34^+^CD38^+^ population was reduced during expansion, ie, ~80% CD34^+^CD38^−^ cells detected after day 5, raising the question of a massive de-differentiation. As it is unlikely that the majority of cells spontaneously de-differentiate during culture, a possible approach was suggested by McKenzie *et al*., where CD38 was described as a reversibly expressed HSC surface marker.^27^

DDR in CD34^+^cells is well studied in the adult counterpart, with other groups showing that adult quiescent CD34^+^ cells, residing in the bone marrow, accumulate DNA damage during aging thus triggering DDR when entering cell cycle.^28^ Biechonski *et al.* emphasize the sensitivity of adult quiescent CD34^+^ cells to DNA damage, in contrast to their progeny, which in combination with the error-prone NHEJ used by the quiescent CD34^+^cells, leads to the drastically impaired regenerative capacity of these cells.^29^

We examined neonatal CD34^+^ cells, whose response to damage still needs to be extensively analyzed due to their clinical application in treatment of hematological malignancies.

### DDR in Cycling CD34^+^ Cells

Cycling CD34^+^ cells were damaged in a dose-dependent manner when faced with distinct genotoxic noxae employing different mode of actions. Cells are sensitive to MNU due to its ability to methylate O^6^-guanine. If the methylation is not removed through direct repair by MGMT or by the several other repair mechanisms, mutations can be accumulated during replication cycles and transferred to progeny. Mutation prevention is thus of great significance for neonatal CD34^+^ cells. Treatment resulted in a high abrogation of cell replication accompanied by the increase of the sub-G_1_ cell fraction and cell cycle stop in a concentration-independent manner until 7 days post-treatment. This comes along with the upregulation of several DDR genes, eg, H2AX, BRCA2, XRCC6, and LIG4 to reduce damage accumulation after treatment with higher MNU concentrations (Supplementary Table S3). The damage increased 5 days after treatment and resulted in the complete cell death of cells treated with > 0.5 mM MNU. This is in accordance with gene expression showing upregulated expression of the pro-apoptotic gene BID and the autophagy-related gene BECN1.

Etoposide is a well-known cytostatic drug, applied to combat a variety of different neoplasms by inducing DSBs in cells through the inhibition of topoisomerase II during replication.^30^ Cycling CD34^+^ cells treated with various concentrations of etoposide (1.5/5/10 µM) indicated different degrees of damage in a dose-dependent manner. Cells treated with 1.5 µM only exhibited signs of proliferation decrease merely directly after treatment. On the other hand, cells treated with both 5 and 10 µM showed different stages of apoptotic morphology, including membrane-bound apoptotic bodies, impaired replication capacity and a cell cycle distribution shift, with an increased sub-G_1_-phase and a decreased S-/G_2_-phase.

Our findings are in accordance with Tao et al., who showed that treatment of cycling HSCs with etoposide leads to apoptosis.^31^ They postulate a DDR response in HSCs, which is tightly controlled by both pro-apoptotic genes, eg, BAX and FAS, and pro-survival genes, eg, BCL-2 and CDKN1A. Their upregulation suggests that, depending on the severity of the genotoxin-induced damage, the balance between the DDR players is altered and thus the fate of the cell determined. The analyzed genes study showed similar regulation upon etoposide exposure, as described by Tao et al., however, eventhough an upregulation of the cell cycle regulators CDKN1A and CCND1 in all conditions was registered, the cell used different DDR mechanisms depending on the applied concentration (Supplementary Table S2). While CDKN1A upregulation is reported to promote cell cycle arrest in both G_1_/S- and G_2_/M-transition to protect the cell from undergoing apoptosis after genotoxic stress,^32^ CCND1 is reported to induce cell cycle re-entry after etoposide-induced cell cycle arrest.^30^ The interplay of these different pro-apoptotic and pro-survival genes along with the analyzed dose-dependent proliferation halt and cell-cycle arrest, leads to the assumption that, while cycling CD34^+^cells treated with etoposide were damaged in a dose-dependent manner, at the time of damage, not all cells were actively cycling. Individual cells were still quiescent or predominantly in the G_1_-phase at the time of damage. The initial response of CD34^+^cells to damage is a CDKN1A-induced cell cycle arrest, whereby the response rapidly shifts to the side of the pro-apoptotic players leading to apoptosis. The alleged recovery of cycling cells after treatment with etoposide can be explained by the fact that at the time of damage a small cell fraction was still quiescent and thus protected from the toxin. These CD34^+^cells could then start proliferation after toxin removal and could thus replenish the HSC pool in the culture. However, analysis of DSB markers γH2AX/53BP1 revealed that all conditions had severe numbers of DSBs accumulated in nearly all cells. In comparison to the extensively studied effect of irradiation on γH2AX/53BP1-foci formation with fast kinetics directly after exposure (1-3 minutes) and a disappearance of foci after 24-48 h,^33^ etoposide treatment led to ambiguous results. With a 1 h delay in foci formation, all conditions displayed similar numbers of γH2AX/53BP1-foci, which did not persist for more than 24 h. This rapid loss of foci leads to the question whether DSBs have been repaired or cells appointed the apoptotic pathway to reduce damage in all conditions. The accurate interplay between γH2AX/53BP1 might also play an important role as we have found cells with only partially co-localized foci. De Feraudy *et al.* studied DSB response in human fibroblasts and suggested that a great number of cells do not display γH2AX/53BP1 co-localization postulating that γH2AX-foci alone might not be an accurate marker of DSBs. This leads to the assumption that only in cells where both foci are present, DDR leads to accurate repair of DSBs.^34^

### DDR in Quiescent CD34^+^ Cells

Quiescent CD34^+^ cells have inherently different DDR mechanisms when faced with different genotoxic noxae. They may apply to either repair damage or protect the cell from transferring mutations to progeny by undergoing apoptosis. It was found that cell cycle statuses protect the cell differently from genotoxins depending on their mode of action. In recent years, nitrosamines have increasingly become the focus of research due to their potential carcinogenic effects. They have also been found to pass the placental barrier, thus possibly posing a threat to neonatal HSCs,^35^ evoking the necessity to study their effect on cord blood HSCs. Quiescent CD34^+^cells are sensitive to MNU treatment, in a dose-dependent manner. Proliferation and further cell cycle progression is affected, with cells being arrested in G_1_ phase and predominantly undergoing apoptosis upon progression to S-phase when treated with high doses of MNU (0.5 mM) to stop the transfer of acquired mutations to progeny. This increase in apoptotic levels is in accordance with the upregulation of pro-apoptotic genes, eg, CAS3, CAS9, and FAS (Supplementary Table S5). In contrast, quiescent CD34^+^cells are mostly protected from cell cycle-specific agents, eg, etoposide, as it is believed that only cells that entered the cell cycle during the 24 h treatment would be affected. However, no significant damage of quiescent CD34^+^cells was observed in terms of morphology and cell counts. Apoptosis assay revealed a small dose-dependent increase in apoptotic cells, which is in accordance with the dose-dependent upregulation of CDKN1A and FAS in all conditions, thus explaining the cell cycle arrest and promotion of apoptosis (Supplementary Table S4). However, the immunocytochemical staining of γH2AX/53BP1-foci revealed the induction of DSBs for all concentrations of etoposide tested, with high numbers of γH2AX-foci detected, with only half as many 53BP1-foci. This raises again the question for further analyzes of DDR-specific genes on a protein basis to verify if repair mechanisms are successfully enacted only in cells with γH2AX/53BP1 co-localization.^34^ The high number of γH2AX-single-foci might be an indicator, that once a DSB is detected in quiescent cells, the apoptotic pathway is directly activated, rather than further repair mechanisms, as it has been shown that non-chromatin-associated γH2AX sensitizes cells to undergo apoptosis, leading to the assumption that γH2AX might not only be a DSB-marker but also an apoptosis-related marker.^36^

Our findings suggest that neonatal CD34^+^cells similarly to their adult counterpart, have elaborated response mechanisms to DNA damage, which operate on a fine balance between pro-apoptotic and pro-survival signals. In contrast to adult CD34^+^ cells which repair accumulated damage after cell cycle entry, both quiescent and cycling neonatal CD34^+^ cells favor the apoptotic cell fate to prevent mutation accumulation and passaging to progeny especially in the case of persistent methylation damage. Nevertheless, the quiescent state of neonatal CD34^+^ cells greatly protects cells from toxins especially those operating in a cell cycle-dependent manner, thus allowing CD34^+^pool replenishment starting from the undamaged quiescent cell.

## Supplementary Material

sxad085_suppl_Supplementary_Figures_S1-S15_Tables_S1-S5

## Data Availability

The data supporting the findings of this study are available from the corresponding author upon reasonable request.
