# Supplementary material for: DNA Damage Response After Treatment of Cycling and Quiescent Cord Blood Hematopoietic Stem Cells With Distinct Genotoxic Noxae
Source: Stem Cells. 2023 Nov 14;42(2):158–71. doi: 10.1093/stmcls/sxad085 (PMC10852021; doi:10.1093/stmcls/sxad085)
Supplement: sxad085_suppl_Supplementary_Figures_S1-S15_Tables_S1-S5 [file sxad085_suppl_supplementary_figures_s1-s15_tables_s1-s5.docx]

**Supporting Information**

**
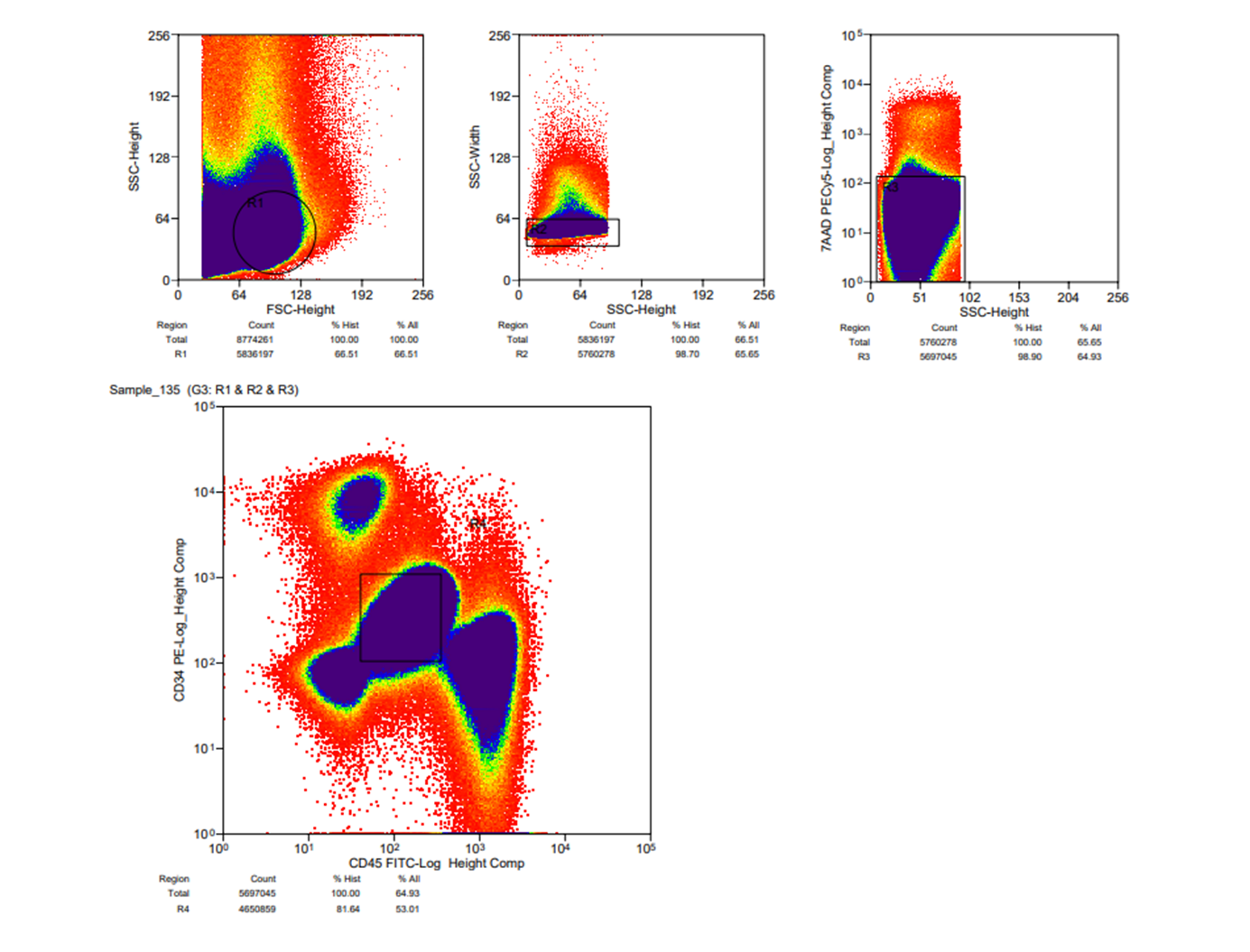
**

Figure S1: Sorting strategy of CD34^+^ CD45^+low^ quiescent HSCs after CliniMACS separation prior to MNU treatment. The figure shows representative scatterplots.


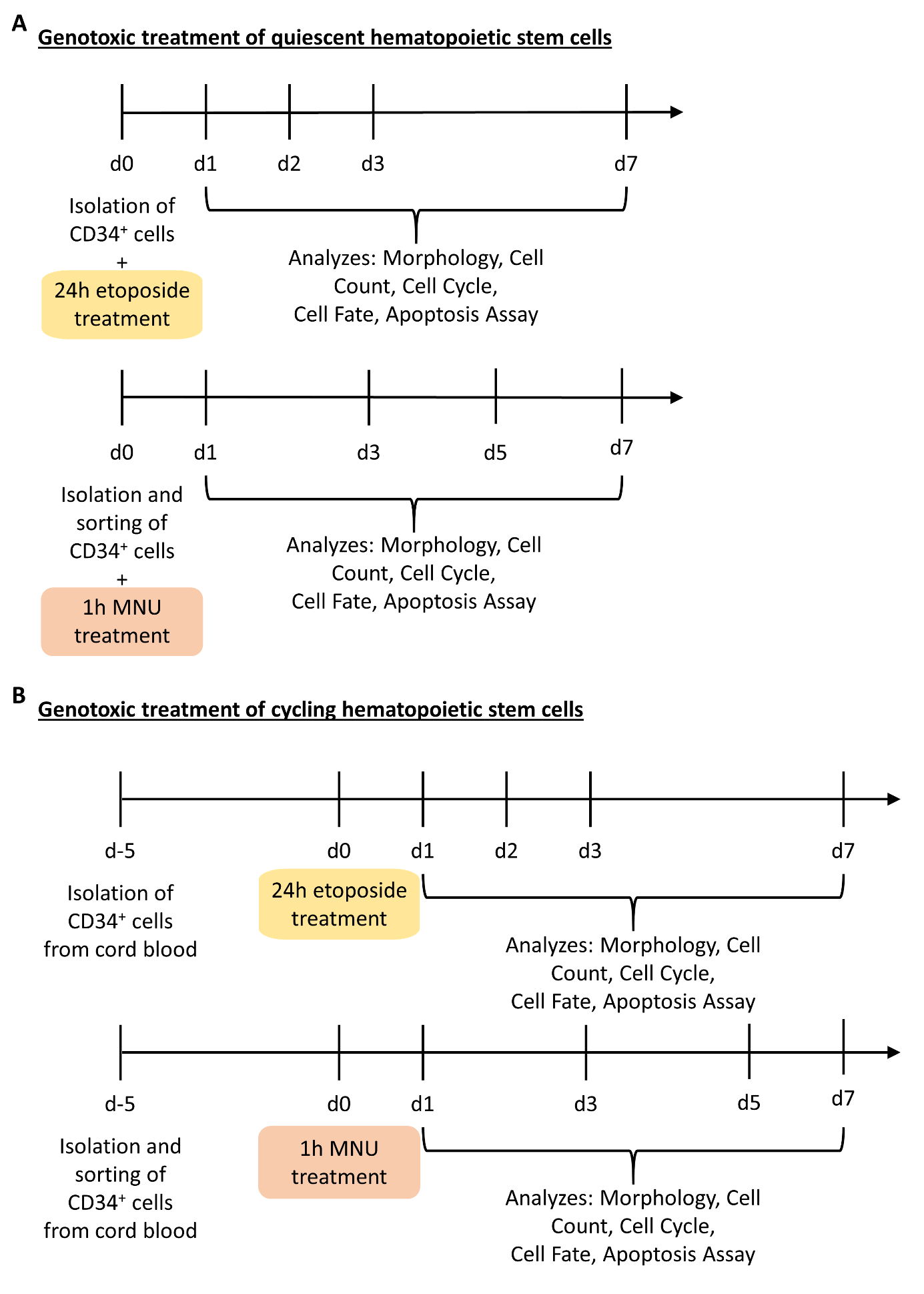


Figure S2: Schematic overview of the experimental setup of genotoxic treatment of quiescent and cycling CD34^+^ cells isolated from cord blood. (A) Quiescent HSCs were isolated from cord blood and directly treated with etoposide (24h) or MNU (1h). Analyzes were performed starting from d1. (B) Cycling HSCs were cultivated for 5 days after isolation from cord blood before treatment with etoposide (24h) or MNU (1h). Analyzes were performed starting from d1. Abbreviations: MNU, N-methyl-N-nitrosurea.


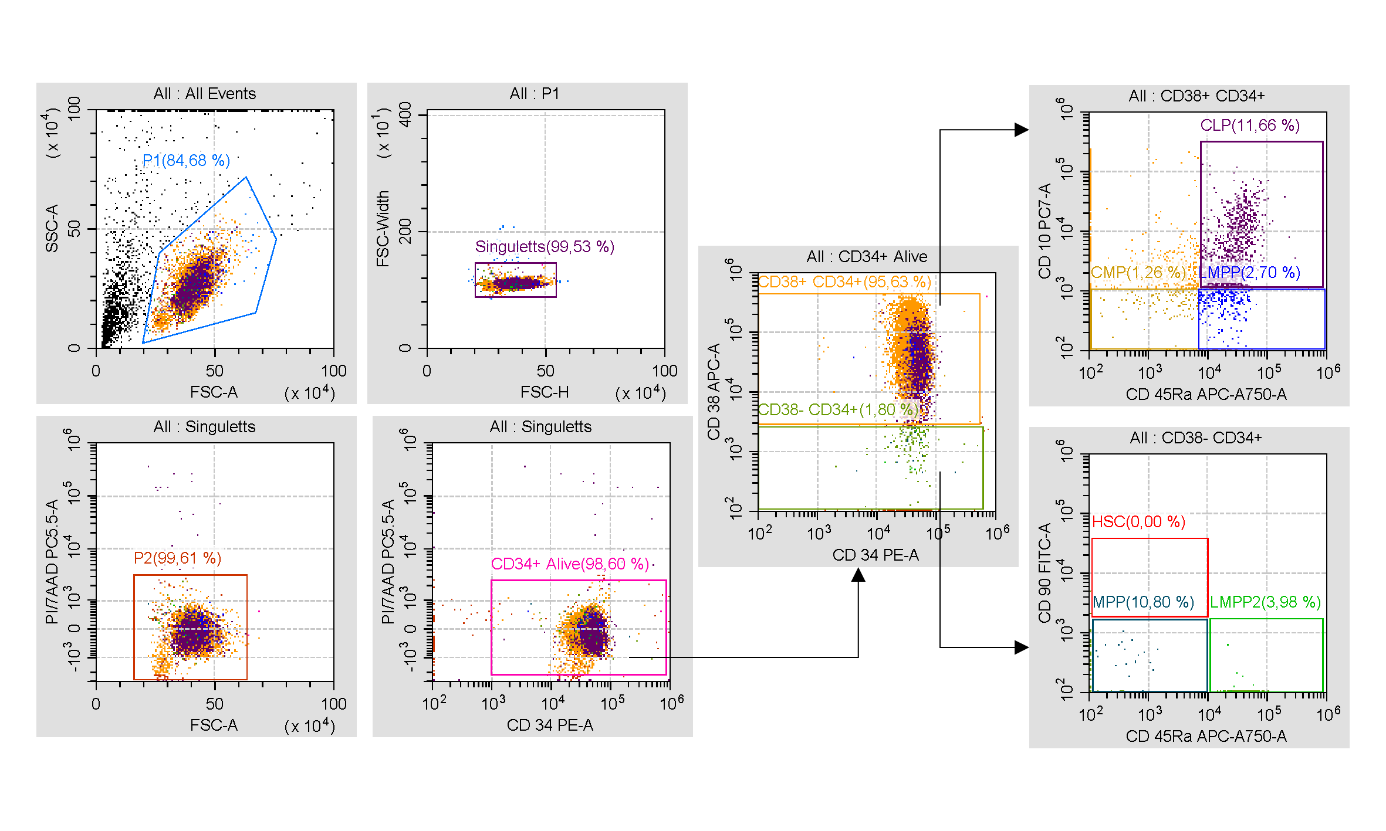


Figure S3: Gating strategy used for flow cytometric cell fate analysis. Alive cells were determined using PI staining (P2). Based on P2, the alive CD34^+^ population was determined. CD38^+^ CD34^+^ and CD38^-^ CD34^+^ populations were distinguished based on the FMO CD38 control. CLP (CD10^+^ CD45Ra^+^), CMP (CD10^-^ CD45Ra^-^) and LMPP (CD10^-^ CD45Ra^+^) were determined based on the CD38^+^ CD34^+^ population. HSC (CD90^+^ CD45Ra^-^), MPP (CD90^-^ CD45Ra^-^) and LMPP (CD90^-^ CD45Ra^+^) were determined based on the CD38^-^ CD34^+^ population. Abbreviations: CLP, common lymphoid progenitor; CMP, common myeloid progenitor; LMPP, lymphomyeloid-primed progenitor; FMO, fluorescence minus one; HSC, hematopoietic stem cell; MPP, multipotent progenitor.


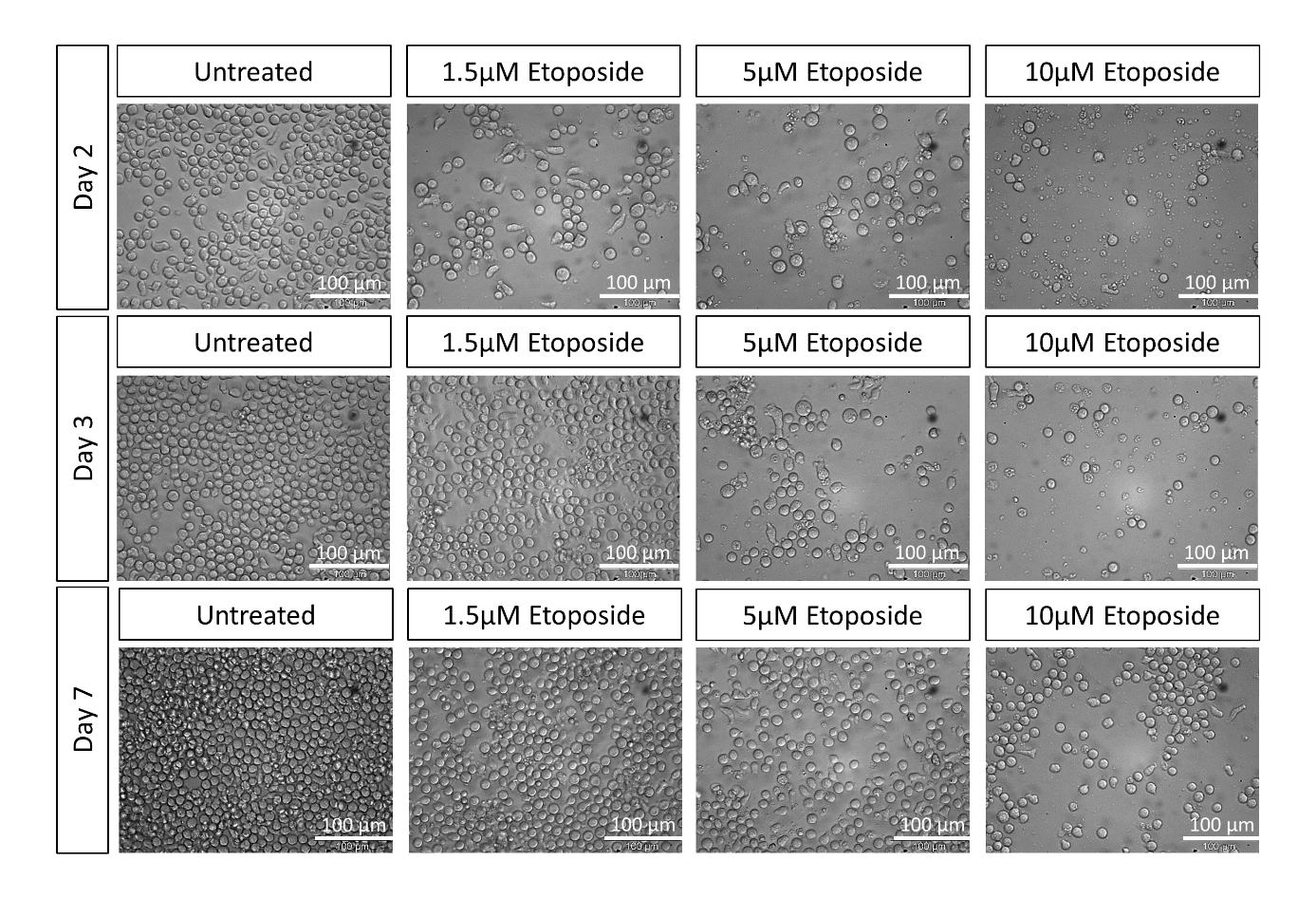


Figure S4: Representative morphological images of cycling CD34^+^ cells treated for 2 h with different etoposide concentrations (1.5µM, 5µM, and 10µM) in comparison to untreated cells. Changes were documented on day 2, 3 and 7 after the start of treatment. Scale bar = 100µm.


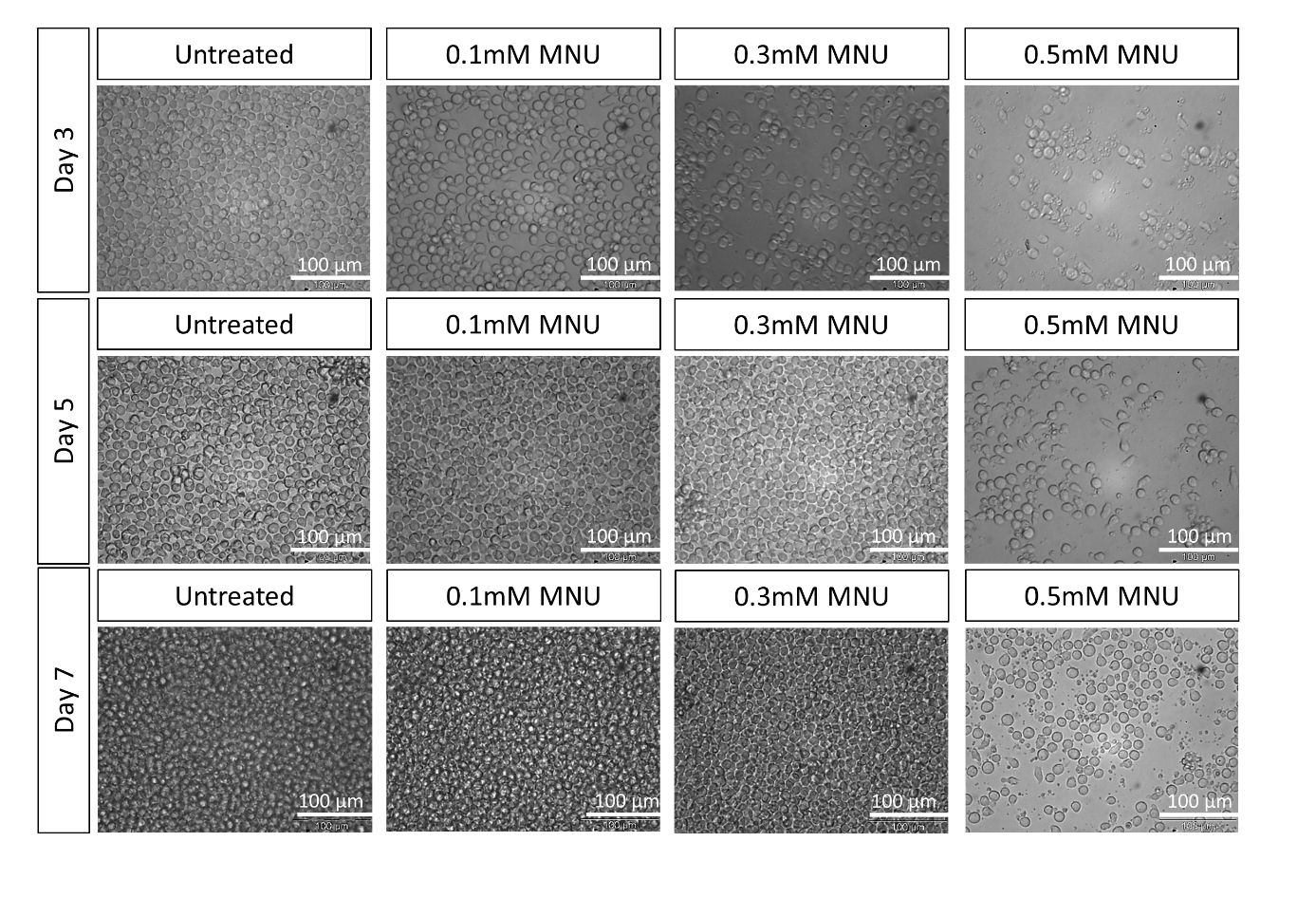


*Figure S5: Representative morphological images of cycling CD34^+^ cells treated for 1h with different MNU concentrations (0.1mM, 0.3mM, and 0.5mM) in comparison to untreated cells. Changes were documented on day 3 and 5 after the start of treatment. Scale bar = 100µm. Abbreviations: MNU, N-methyl-N-nitrosurea.*


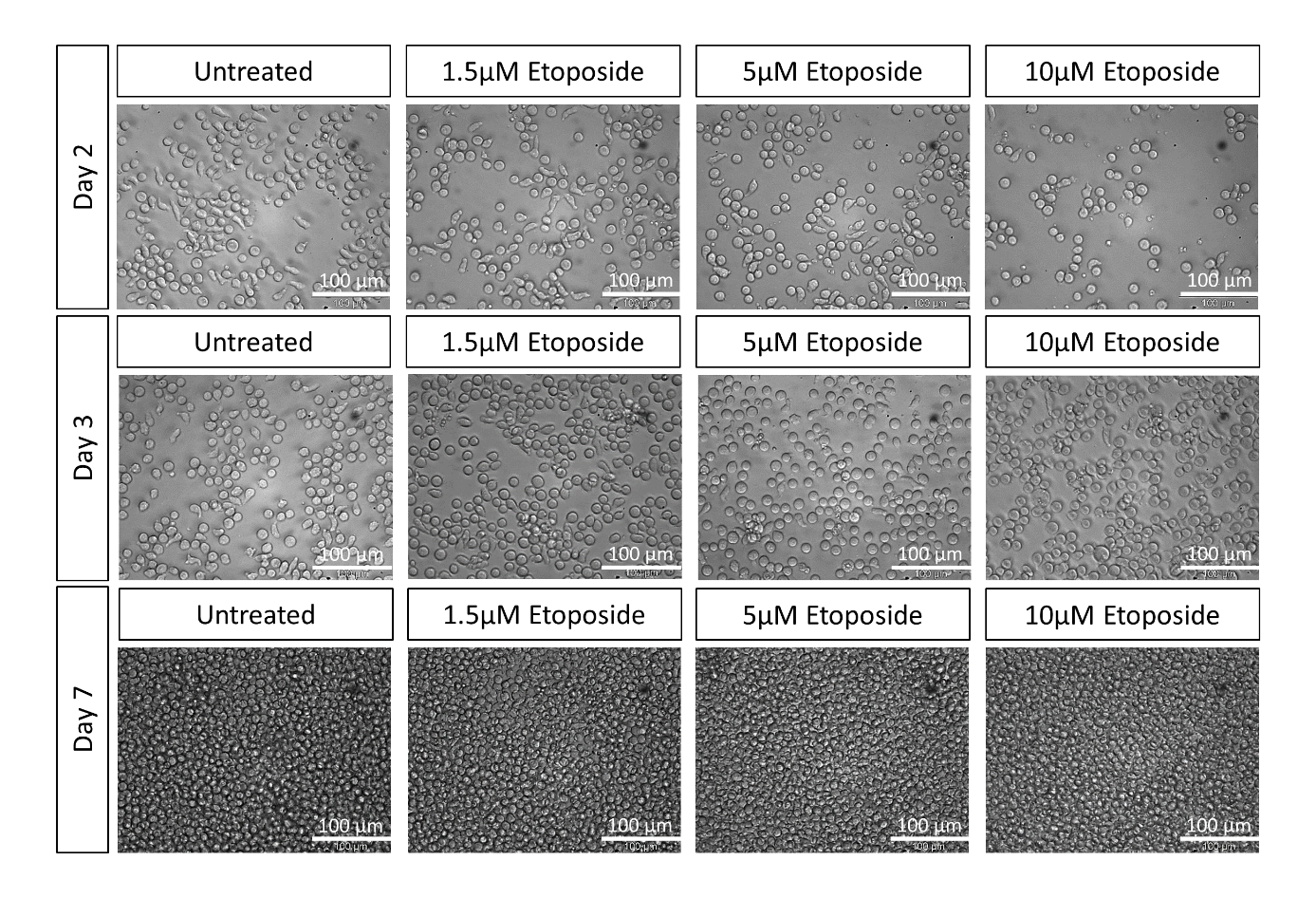


Figure S6: Representative morphological images of quiescent CD34^+^ cells treated for 24h with different etoposide concentrations (1.5µM, 5µM, and 10µM) in comparison to untreated cells. Changes were documented on day 2, 3 and 7 after the start of treatment. Scale bar = 100µm.

*
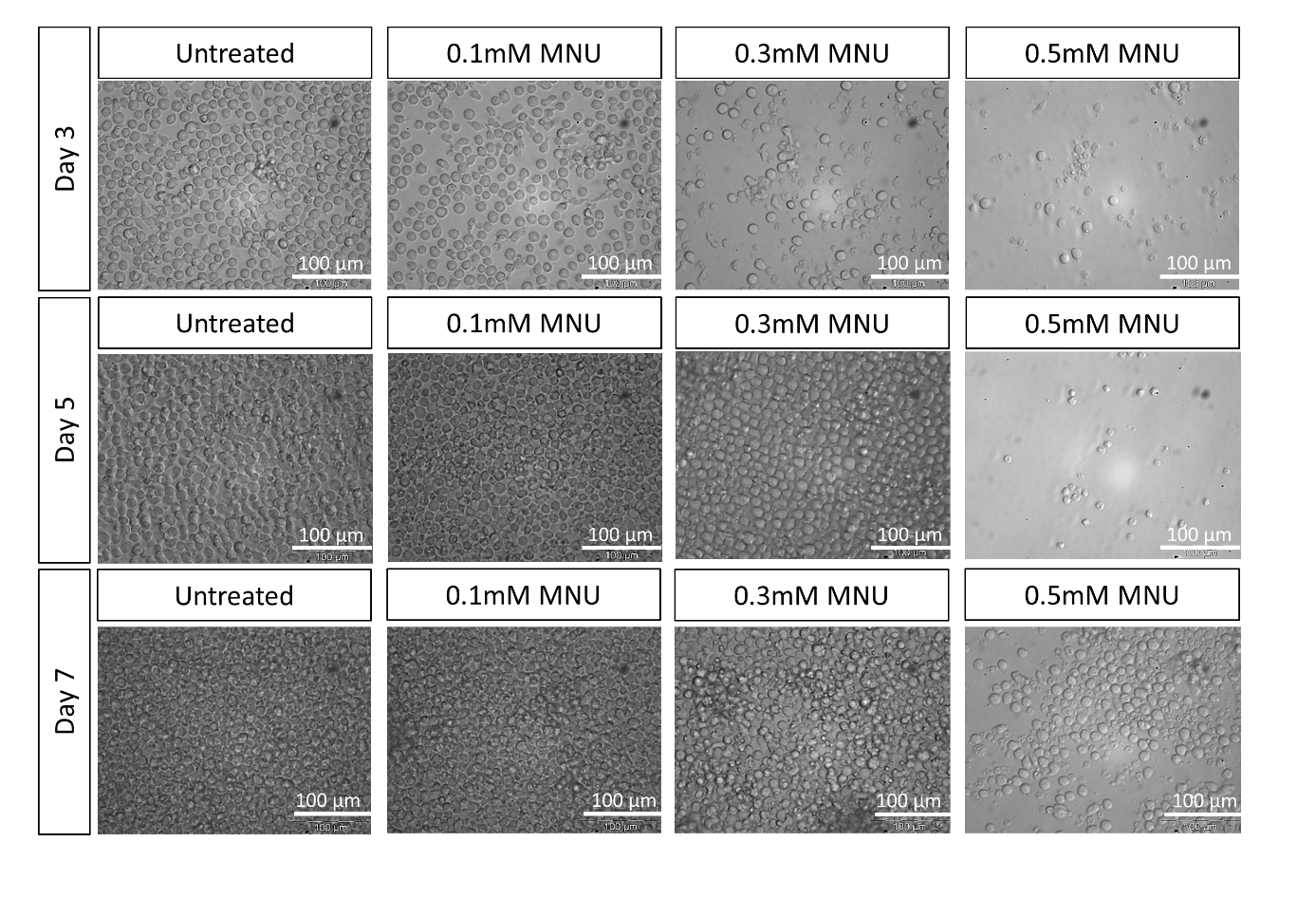
Figure S7: Representative morphological images of quiescent CD34^+^ cells treated for 1h with different MNU concentrations (0.1mM, 0.3mM, and 0.5mM) in comparison to untreated cells. Changes were documented on day 3 and 5 after the start of treatment. Scale bar = 100µm. Abbreviations: MNU, N-methyl-N-nitrosurea.*

*
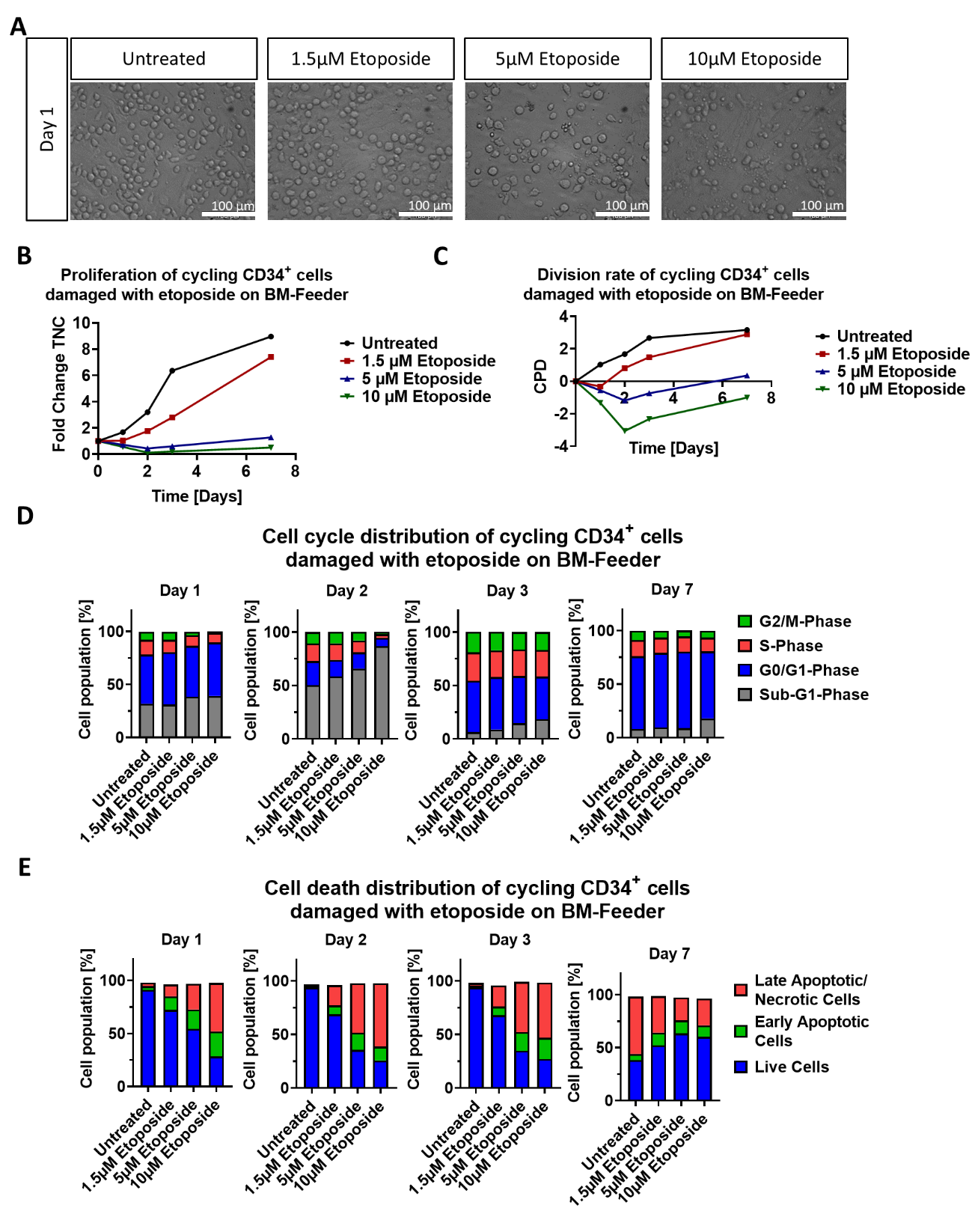
*

*Figure S8: Effect of etoposide treatment on morphology, growth, cell cycle and cell death of cycling CD34^+^ cell on BM-Feeder. (A) Representative morphological images of untreated and with 1.5µM, 5µM and 10µM etoposide treated cycling CD34^+^ cells. Analysis was performed after a 24h treatment. Scale bars = 100µm. (B, C) Representative growth kinetics depicted via the total fold change over time and the CPD. After treatment of cycling cells with different etoposide concentrations, the growth curves revealed no significant effect on cycling CD34^+^ cells. (D) Cell cycle analysis of treated cycling CD34^+^ cells via staining with PI and analysis by flow cytometry. (E) Analysis of cell death levels of treated cycling CD34^+^ cells via Annexin V/PI staining. (B-E) Shown are representative data from three independent experiments with different CB donations. Abbreviations: BM-Feeder, bone marrow feeder; CB, cord blood; CPD, cumulative population doubling, PI, propidium iodide.*


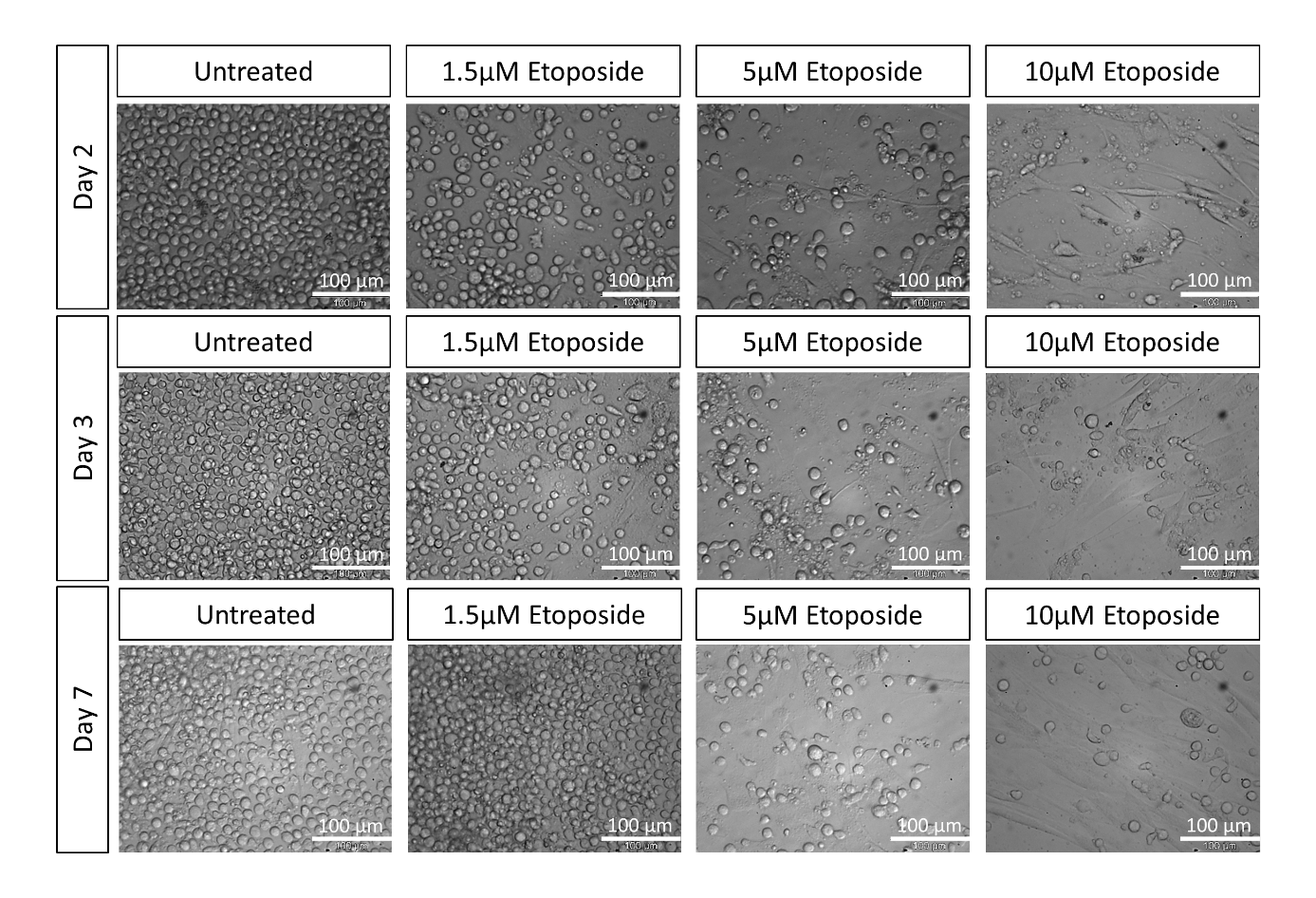


*Figure S9: Representative morphological images of cycling CD34^+^ cells treated for 1h with different etoposide concentrations (1.5µM, 5µM, and 10µM) on BM-Feeder in comparison to untreated cells. Changes were documented on day 2, 5 and 7 after the start of treatment. Scale bar = 100µm.*


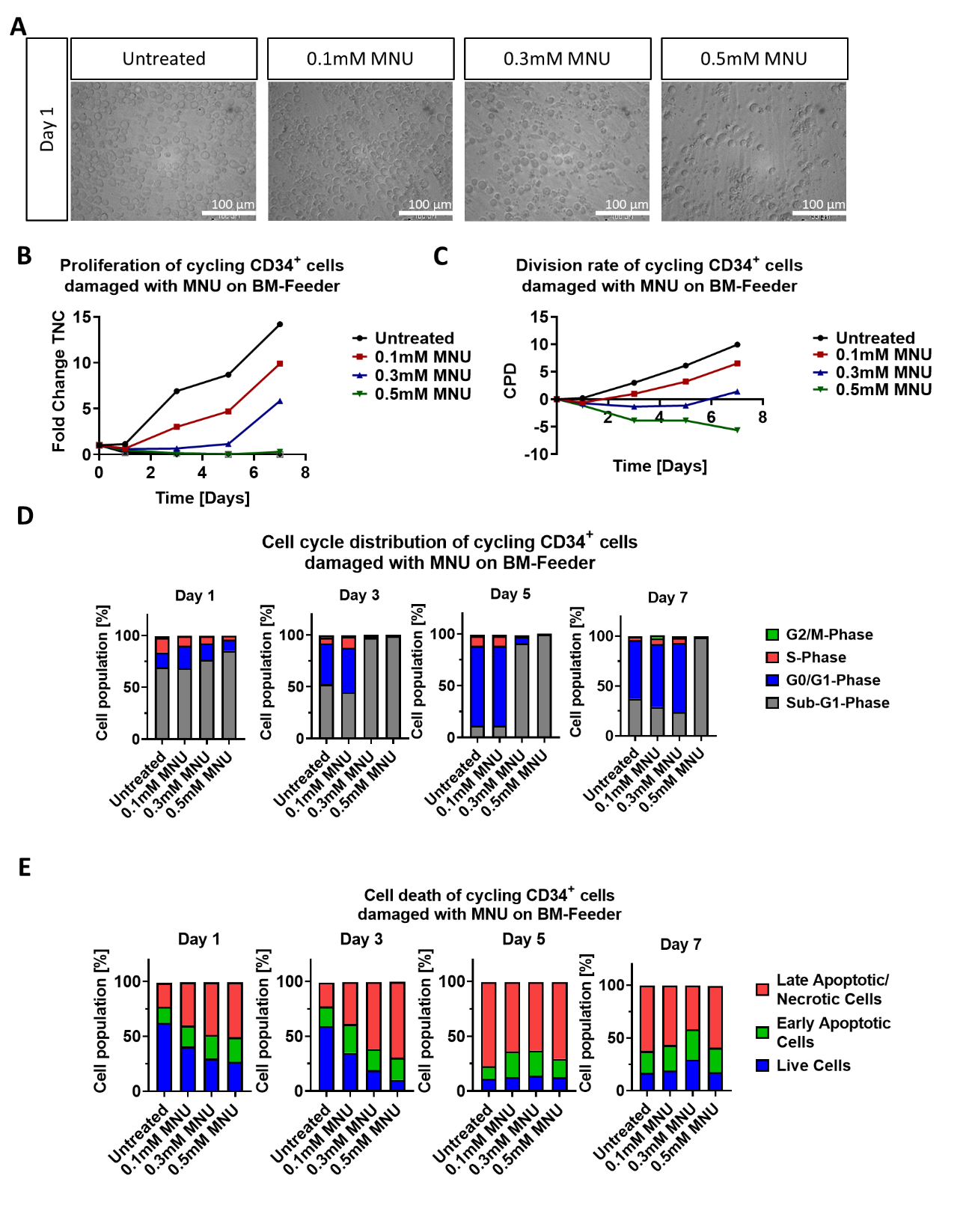


*Figure S10: Effect of MNU treatment on morphology, growth, cell cycle and cell death of cycling CD34^+^ cell on BM-Feeder. (A) Representative morphological images of untreated and with 0.1mM, 0.3mM and 0.5mM MNU treated cycling CD34^+^ cells. Analysis was performed after a 1h treatment. Scale bars = 100µm. (B, C) Representative growth kinetics depicted via the total fold change over time and the CPD. After treatment of cycling cells with different MNU concentrations, the growth curves revealed no significant effect on cycling CD34^+^ cells. (D) Cell cycle analysis of treated cycling CD34^+^ cells via staining with PI and analysis by flow cytometry. (E) Analysis of cell death levels of treated cycling CD34^+^ cells via Annexin V/PI staining. (B-E) Shown are representative data from three independent experiments with different CB donations. Abbreviations: BM-Feeder, bone marrow feeder; CB, cord blood; CPD, cumulative population doubling, MNU, N-methyl-N-nitrosurea; PI, propidium iodide.*

*
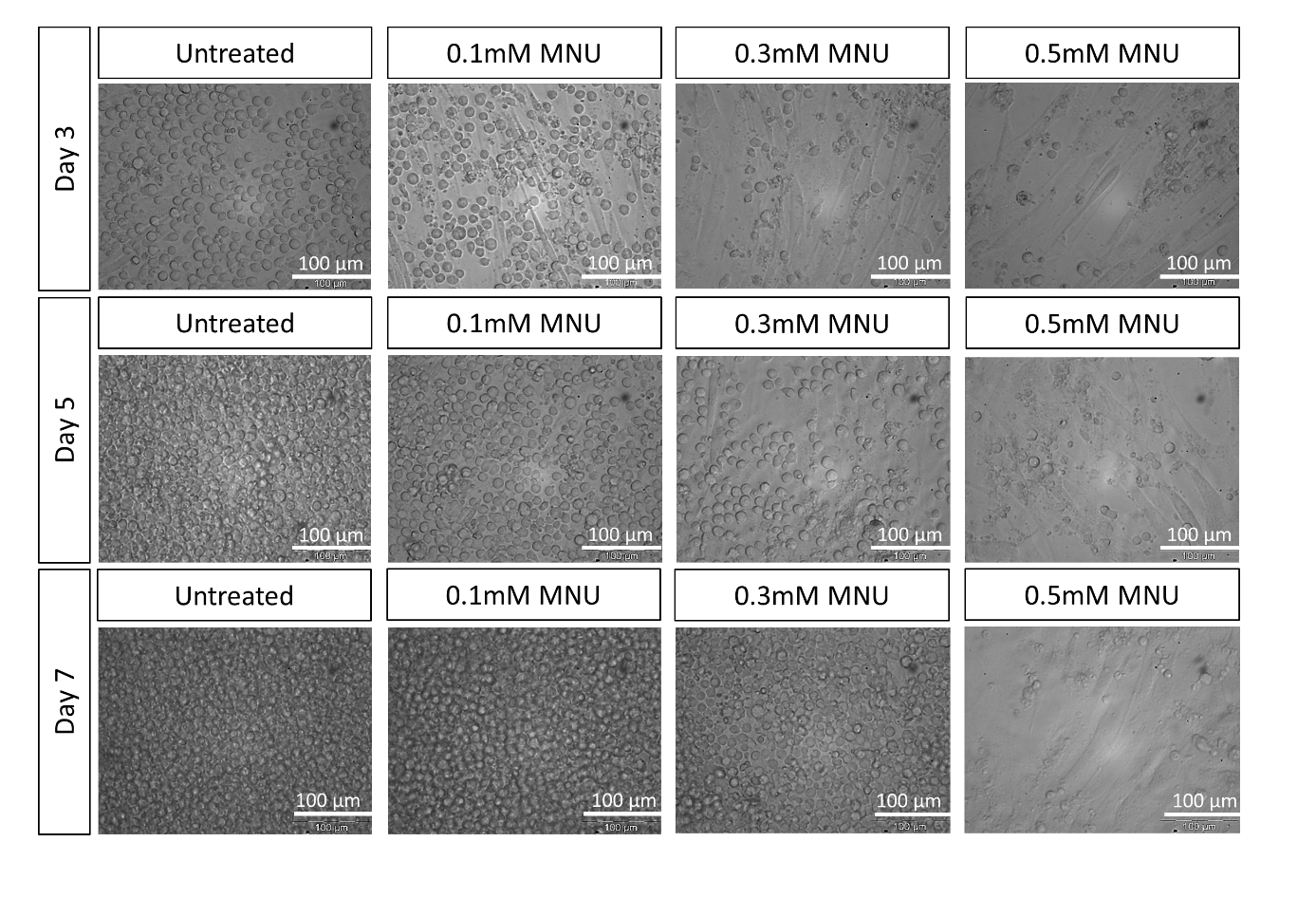
Figure S11: Representative morphological images of cycling CD34^+^ cells treated for 1h with different MNU concentrations (0.1mM, 0.3mM, and 0.5mM) on BM-Feeder in comparison to untreated cells. Changes were documented on day 3, 5 and 7 after the start of treatment. Scale bar = 100µm. Abbreviations: MNU, N-methyl-N-nitrosurea.*


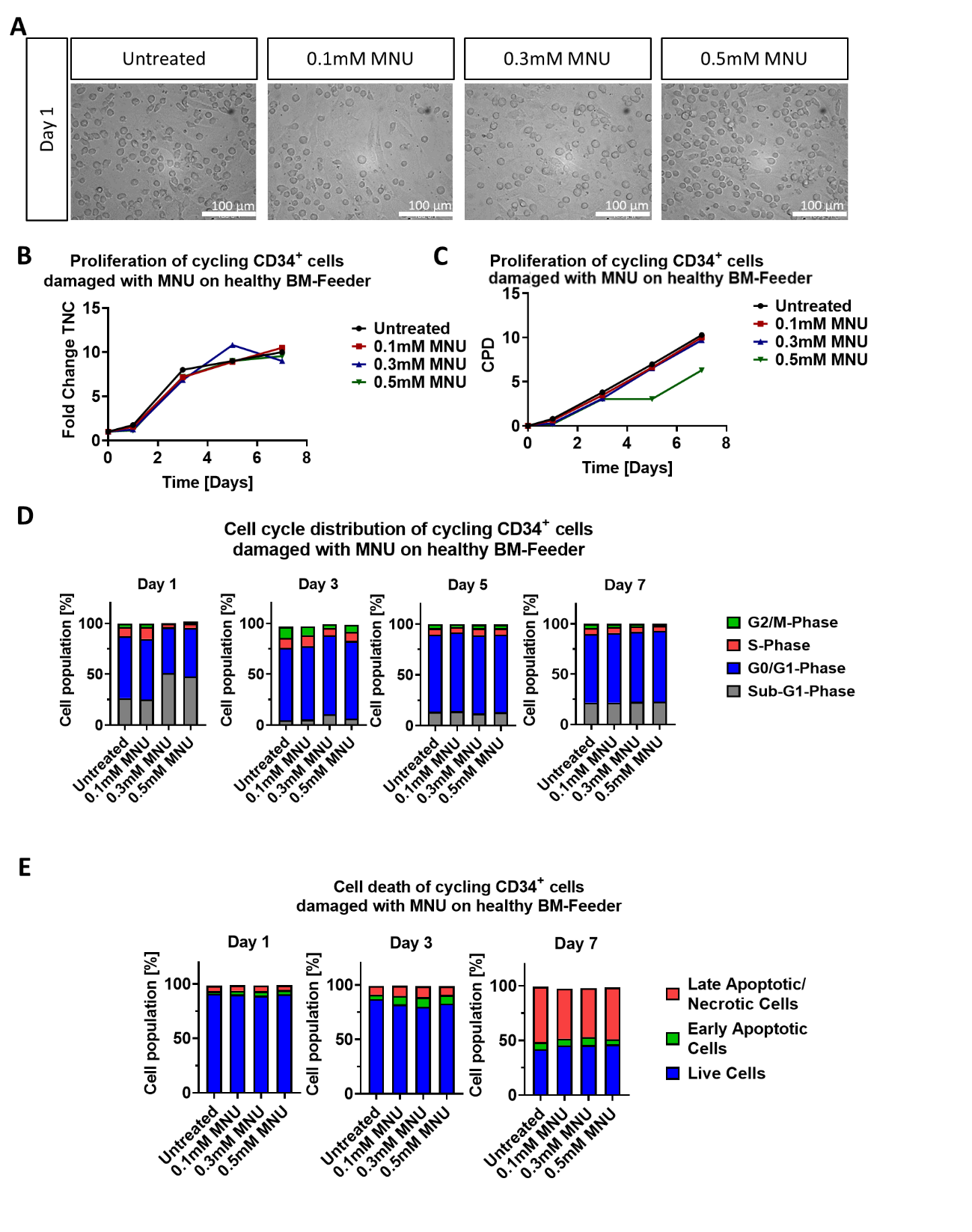


*Figure S12: Effect of MNU treatment on morphology, growth, cell cycle and cell death of cycling CD34^+^ cell on healthy BM-Feeder. (A) Representative morphological images of untreated and with 0.1mM, 0.3mM and 0.5mM MNU treated cycling CD34^+^ cells. Analysis was performed after a 1h treatment. Scale bars = 100µm. (B, C) Representative growth kinetics depicted via the total fold change over time and the CPD. After treatment of cycling cells with different MNU concentrations, the growth curves revealed no significant effect on cycling CD34^+^ cells. (D) Cell cycle analysis of treated cycling CD34^+^ cells via staining with PI and analysis by flow cytometry. (E) Analysis of cell death levels of treated cycling CD34^+^ cells via Annexin V/PI staining. (B-E) Shown are representative data from three independent experiments with different CB donations. Abbreviations: BM-Feeder, bone marrow feeder; CB, cord blood; CPD, cumulative population doubling, MNU, N-methyl-N-nitrosurea; PI, propidium iodide.*


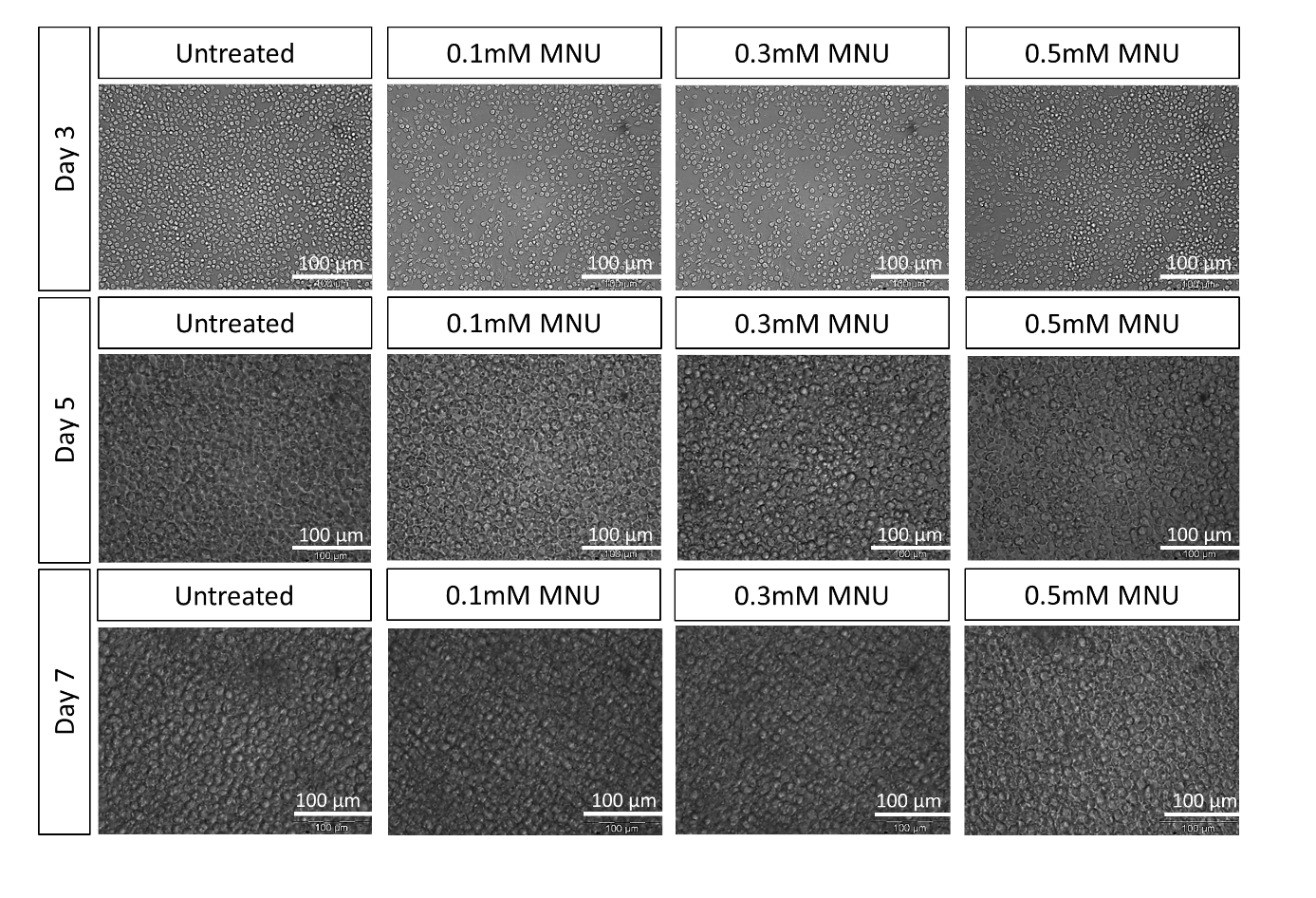


*Figure S13: Representative morphological images of cycling CD34^+^ cells treated for 1h with different MNU concentrations (0.1mM, 0.3mM, and 0.5mM) and cultivated on healthy BM-Feeder in comparison to untreated cells. Changes were documented on day 3, 5 and 7 after the start of treatment. Scale bar = 100µm. Abbreviations: MNU, N-methyl-N-nitrosurea.*


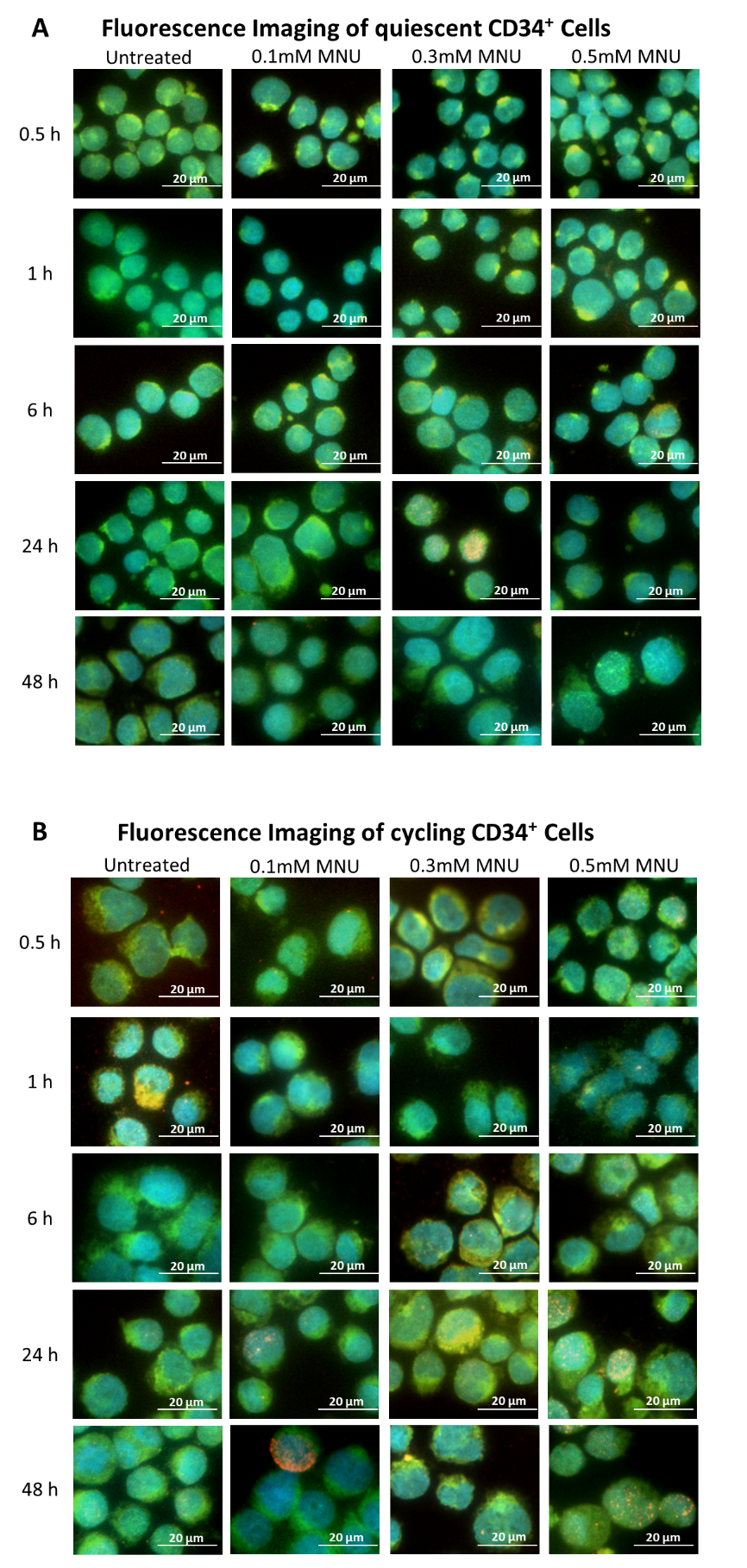


*Figure S14: Representative fluorescence co-staining images of (A) quiescent CD34+ cells treated with different doses of MNU, and (B) cycling CD34+ cells treated with different doses of MNU. Cells were treated with 0.1mM, 0.3mM, or 0.5mM MNU, and harvested between 0.5h and 48h and co-stained for γH2AX (green) and 53BP1 (red), using HOECHST (blue) to visualize the nucleus. Scale bars = 20 µm.*


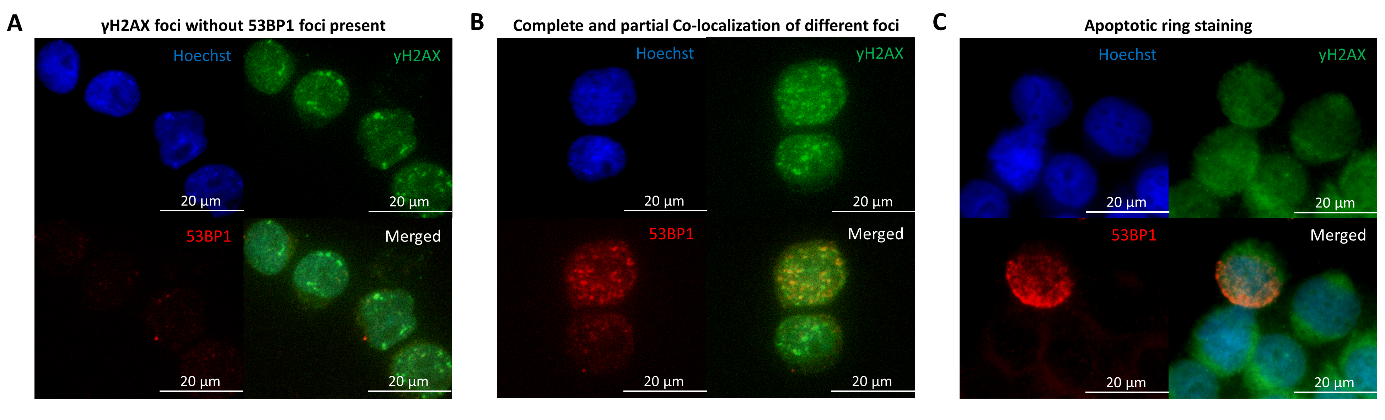


*Figure S15: Representative fluorescent images of γH2AX and 53BP1 co-localization staining. CD34^+^ cells were treated with different genotoxic noxae (etoposide or MNU) and cells stained with γH2AX (green) and 53BP1 (red), using HOECHST (blue) to visualize the nucleus. Distinct γH2AX/53BP1 staining types were detected. These different types of foci were (A) only γH2AX with no 53BP1 foci present, (B) partial and complete co-localization of the γH2AX- and 53BP1-foci in a cell, and (C) apoptotic rings, where staining of 53BP1 revealed staining of the circuit of cells. Scale bars = 20 µm.*

Table S1: List of utilized qRT-PCR primers. All primer sequences are listed from 5´ to 3´. All primers were obtained from Thermo Fisher Scientific.

| **Gene** | **Forward primer (5´-3´)** | **Reverse primer (5´-3´)** |
| --- | --- | --- |
| *APEX1* | *ACAAAGAGGCAGCAGGAGAG* | *CTGGGGCTTCTTCCTTTACC* |
| *ATG3* | *AGGACAATATAAGGCTTCAA* | *TTCCAACAATCCACTCTC* |
| *ATM* | *CCTTGTGCTAGTGGGCAGAA* | *ATGGGGAGCAAAGAACCCAG* |
| *BAX* | *CGGGGAGCAGCCCAG* | *GGAGTCTGTGTCCACGGC* |
| *BCL-2* | *GAACTGGGGGAGGATTGTGG* | *CCGTACAGTTCCACAAAGGC* |
| *BECN1* | *GGATGGAAGGGTCTAAGA* | *CTGTGGTAAGTAATGGAG* |
| *BID* | *GGGAAACTGTTGAGTGGCTGA* | *CAGCTCCGACTCACTCCTG* |
| *BRCA1* | *CCACAGATCAACTGGAATGG* | *GTAGAGTGCTACACTGTCCA* |
| *BRCA2* | *TTCTGAGGTGGACCTAATAGG* | *TGATTTGGATTCTGGTCGCC* |
| *CAS3* | *AGCAAACCTCAGGGAAACATT* | *CTCAGAAGCACACAAACAAAAC* |
| *CAS8* | *AATGTTGGAGGAAAGCAATC* | *CATAGTCGTTGATTATCTTCAGC* |
| *CAS9* | *AACCCTAGAAAACCTTACCCC* | *CATCACCAAATCCTCCAGAAC* |
| *CCNB1* | *GCATCTAAGATTGGAGAGGTTGAT* | *CAGGTAATGTTGTAGAGTTGGTG* |
| *CCND1* | *GATGCCAACCTCCTCAACGA* | *GGAAGCGGTCCAGGTAGTTC* |
| *CDK1* | *AAATTGAGCGGAGAGCGACG* | *TGGCTACCACTTGACCTGTAG* |
| *CDK6* | *TGGAGACCTTCGAGCACC* | *CACTCCAGGCTCTGGAACTT* |
| *CDKN1A* | *TACATCTTCTGCCTTAGT* | *TCTTAGGAACCTCTCATT* |
| *CDKN1B* | *ACAGCTCGAATTAAGAATA* | *CTTATACAGGATGTCCATT* |
| *FAS* | *TGTGTGATGAAGGACATGGCTTA* | *ACTTGGTGTTGCTGGTGAGT* |
| *FANCA* | *CGCAGGTCACGGTTGATGTA* | *CCACTGAACACTCCGAACCA* |
| *FANCI* | *CTGTGATATGAGCAACAATGG* | *TTCACTGCTTGATTCTGAAGG* |
| *H2AX* | *TTCTGGAAGACTTGGCCTTC* | *ACTCTTGCTGTCCACATAGC* |
| *LIG4* | *TTGCCCGAGGCCAGTTAAA* | *CACAAATCTGCAAAAGGAACGTG* |
| *MGMT* | *ACCGTTTGCGACTTGGTACT* | *GGGCTGGTGGAAATAGGCAT* |
| *mKI67* | *TCCTTTGGTGGGCACCTAAGACCTG* | *TGATGGTTGAGGCCTGTTCCTTGATG* |
| *NHEJ1* | *CCATTGTTGAAGGACGCTGC* | *CTAGCTCCCTCACTTGGCAC* |
| *RAD51* | *GCTGATGAGTTTGGTGTAGC* | *AACATAGCTTCAGCTTCAGG* |
| *RPL13a* | *GAGGTATGCTGCCCCACAAA* | *TTCAGACGCACGACCTTGAG* |
| *TP53* | *TTCCGAGAGCTGAATGAGGC* | *AATGTCAGTCTGAGTCAGGCC* |
| *XPC* | *GACCTCAAGAAGGCACACCA* | *TGGCTTCACAGGCAGAAGAG* |
| *XPG* | *TGCACCCCGGTCTTCCATTA* | *CCTCCCTGCTCCTACACAAC* |
| *XRCC1* | *GTCGCCATCTGTTCCCAAGA* | *AAGCCACTCAGCACCACTAC* |
| *XRCC4* | *CCTCAGGAGAATCAGCTTCAAGA* | *AAAGAGGTCTTCTGGGCTGC* |
| *XRCC5* | *AGCATAGACTGCATCCGAGC* | *TCCCCATACATCCACGACCT* |
| *XRCC6* | *TGCGTGGATTGTCGTCTTCT* | *CTTCTTCATCGCCCTCGGTT* |

Table S2: Selection of genes involved in DNA damage repair, cell cycle regulation and apoptosis analyzed in cycling CD34^+^ treated with etoposide (1.5µM, 5µM, and 10µM). Fold changes in mRNA expression were analyzed using qPCR and calculated with the 2-ΔΔCT method relative to untreated CD34^+^ cells and normalized to the reference gene RPL13A. The value 1 corresponds to a basic expression as in untreated cells; Values above 2 are considered a significant upregulation (green) while values below -2 are considered a significant downregulation (red) of a gene in treated CD34^+^ cells. Abbreviations: ETO, Etoposide; HR, Homologous Repair; NHEJ, Non-Homologous End Joining; n.p., not performed.

|  | | **ETO 5h** | | | **ETO 16h** | | | **ETO 24h** | | | **ETO 48h** | | | **ETO 72h** | | |
| --- | --- | --- | --- | --- | --- | --- | --- | --- | --- | --- | --- | --- | --- | --- | --- | --- |
|  |  | 1.5µM ETO | 5µM ETO | 10µM ETO | 1.5µM ETO | 5µM ETO | 10µM ETO | 1.5µM ETO | 5µM ETO | 10µM ETO | 1.5µM ETO | 5µM ETO | 10µM ETO | 1.5µM ETO | 5µM ETO | 10µM ETO |
| **Pathways** | **Gene** | **Fold Change** | | | | | | | | | | | | | | |
| **Cell cycle regulation** | CDKN1A | 3.853 | 17.372 | 17.395 | 8.962 | 27.429 | 27.505 | 10.095 | 30.143 | 53.995 | 16.058 | 30.960 | 54.927 | 13.139 | 11.133 | 18.198 |
|  | CDKN1B | 1.063 | 1.647 | -1.499 | 1.067 | -1.047 | -1.838 | -1.198 | 1.277 | 1.112 | -1.252 | -1.433 | -1.148 | -1.842 | -2.070 | -3.040 |
|  | CCNB1 | -1.164 | -1.563 | -2.793 | 1.009 | -1.188 | -3.802 | 1.140 | -1.073 | -1.883 | -1.043 | -1.294 | -3.155 | 1.178 | -1.319 | -2.857 |
|  | CCND1 | 1.128 | 1.046 | -1.282 | 1.501 | 1.886 | 1.618 | 3.301 | 13.588 | 5.572 | 3.041 | 4.542 | 2.779 | 6.566 | 7.875 | 4.440 |
|  | CDK1 | -1.179 | -2.899 | -1.704 | 1.103 | -1.130 | -1.661 | 1.200 | 1.302 | 1.073 | -1.214 | -1.189 | -1.063 | 1.071 | -1.314 | -3.759 |
| **Apoptosis** | BAX | 1.298 | 1.283 | 1.757 | 1.446 | 2.267 | 2.253 | 2.158 | 2.530 | 2.917 | 2.640 | 2.304 | 2.652 | 4.272 | 3.440 | 4.316 |
|  | BCL-2 | 1.123 | -1.270 | -1.426 | -1.017 | 1.048 | -1.398 | -1.022 | 1.034 | -1.120 | -1.462 | -1.454 | -1.279 | -1.117 | -1.409 | -1.881 |
|  | CAS3 | 1.182 | -1.081 | -1.171 | -1.123 | -1.391 | -3.373 | -1.152 | -1.496 | -1.972 | -1.582 | -1.439 | -2.090 | -1.054 | -1.243 | -2.237 |
|  | CAS9 | 1.228 | 1.012 | -1.173 | 1.005 | 1.013 | -1.848 | -1.034 | -1.162 | -1.278 | -1.384 | -1.292 | -1.021 | 1.091 | 1.048 | 1.176 |
|  | FAS | 2.686 | 5.347 | 5.266 | 3.488 | 5.770 | 3.204 | 3.515 | 5.220 | 5.874 | 2.507 | 3.909 | 5.240 | 3.846 | 4.644 | 2.159 |
| **NHEJ** | XRCC6 | 1.252 | 1.183 | -1.200 | -1.253 | -1.515 | -3.205 | -1.131 | -1.435 | -1.634 | -1.610 | -2.024 | -1.745 | -1.089 | -1.890 | -3.086 |
|  | XRCC4 | 1.138 | 1.046 | -1.181 | -1.181 | -1.245 | -2.079 | 1.049 | -1.155 | -1.493 | -1.048 | -1.466 | -1.404 | -1.130 | -1.748 | -2.151 |
|  | XRCC5 | 1.115 | -1.218 | -1.081 | -1.385 | -1.508 | -2.519 | -1.076 | -1.456 | -1.560 | -1.464 | -1.675 | -1.441 | -1.035 | -1.435 | -1.302 |
|  | NHEJ1 | 1.134 | -1.106 | -1.292 | -1.284 | -1.414 | -2.778 | -1.071 | -1.300 | -1.377 | -1.094 | -1.333 | -1.357 | 1.057 | -1.486 | -1.898 |
|  | LIG4 | 1.316 | 1.109 | -1.408 | -1.449 | -1.464 | -2.037 | -1.114 | 1.122 | -1.030 | 1.021 | -1.332 | 1.209 | 1.734 | 1.484 | 1.172 |
| **HR** | RAD51 | -1.166 | -1.290 | -1.142 | 1.078 | 1.089 | 1.053 | 1.051 | -1.065 | -1.018 | 1.439 | -1.499 | -2.123 | 1.069 | -1.004 | -1.002 |
|  | BRCA1 | -1.124 | -1.068 | -1.086 | 1.022 | 1.144 | -1.401 | -1.120 | -1.311 | -1.074 | 1.352 | -1.502 | -2.294 | -1.003 | 1.014 | 1.220 |
|  | BRCA2 | -1.209 | -1.350 | -1.203 | 1.036 | -1.044 | -1.024 | -1.125 | -1.088 | 1.189 | 1.186 | -1.323 | -2.110 | 1.045 | -1.073 | 1.237 |
| **Involved in DDR** | ATM | -1.170 | -1.433 | 1.088 | -1.319 | -1.387 | -1.626 | -1.795 | -1.608 | -1.486 | -1.727 | -1.842 | -1.957 | -1.449 | -1.351 | -1.376 |
|  | TP53 | 1.142 | 1.663 | -1.171 | -1.120 | -1.082 | -2.119 | -1.134 | -1.351 | -1.592 | -1.812 | -1.626 | -1.490 | -1.637 | -2.208 | -3.003 |
|  | FANCA | 1.095 | -1.065 | 1.032 | -1.092 | -1.040 | -1.055 | -1.410 | -1.171 | -1.092 | 1.185 | -1.949 | -3.257 | -1.675 | -1.379 | -1.166 |

Table S3: Selection of genes involved in DNA damage repair, cell cycle regulation and apoptosis analyzed in cycling CD34^+^ treated with MNU (1mM, 3mM, and 5mM) for 1h. Fold changes in mRNA expression were analyzed using qPCR and calculated with the 2-ΔΔCT method relative to untreated CD34^+^ cells and normalized to the reference gene RPL13A. The value 1 corresponds to a basic expression as in untreated cells; Values above 2 are considered a significant upregulation (green) while values below -2 are considered a significant downregulation (red) of a gene in treated CD34^+^ cells. Abbreviations: HR, Homologous Repair; MNU, N-methyl-N-nitroso-urea; NHEJ, Non-Homologous End Joining; n.p., not performed.

|  | | **MNU d3** | | **MNU d5** | |
| --- | --- | --- | --- | --- | --- |
|  |  | 1mM MNU | 3mM MNU | 1mM MNU | 3mM MNU |
| **Pathways** | **Gene** | **Fold Change** | | | |
| **Direct repair** | MGMT | 1.033 | 1.331 | 1.909 | 2.868 |
| **Cell cycle regulation** | CDKN1A | -1,894 | 1,903 | 3,540 | 6,878 |
|  | CDKN1B | -1.126 | 1.601 | 1.205 | 3.846 |
|  | CCNB1 | -1.226 | -1.403 | -1.353 | 1.805 |
|  | CCND1 | -1.249 | 1.382 | -1.612 | 1.466 |
| **Apoptosis** | BAX | 1.061 | 2.000 | 1.336 | 5.039 |
|  | BCL-2 | -1.303 | -1.216 | -1.658 | 1.715 |
|  | CAS3 | 1.082 | 1.570 | 1.629 | 11.397 |
|  | CAS9 | -1.354 | 1.469 | n.p. | n.p. |
|  | CAS8 | -1.473 | -1.165 | 1.460 | 1.958 |
|  | FAS | -2.490 | 1.019 | 1.281 | 10.027 |
| **NHEJ** | XRCC6 | 1.507 | 3.374 | 2.730 | 29.396 |
|  | XRCC4 | 1.356 | 1.241 | 2.017 | 5.186 |
|  | NHEJ1 | -2.658 | -1.414 | 2.316 | 15.138 |
|  | LIG4 | 1.716 | 3.278 | -2.351 | 1.688 |
| **HR** | RAD51 | 1.122 | 1.901 | -1.251 | 2.378 |
|  | BRCA2 | 2.109 | 3.931 | 3.815 | 13.061 |
| **Autophagy** | BID | 1.863 | 3.014 | n.p. | n.p. |
|  | ATG3 | 1.594 | 2.110 | 1.803 | 8.467 |
|  | BECN1 | 1.130 | 2.758 | n.p. | n.p. |
| **NER** | XPC | n.p. | n.p. | 1,500 | 3,810 |
|  | XPG | n.p. | n.p. | 1.737 | 4.365 |
| **Quiescence** | mKi67 | n.p. | n.p. | 1.521 | 12.985 |
|  | CDK6 | n.p. | n.p. | 1.431 | 10.572 |
| **Involved in DDR** | H2AX | 2.348 | 4.295 | 1.043 | 3.493 |
|  | TP53 | 1.144 | 1.666 | 3.552 | 12.585 |
|  | APEX1 | -3.681 | -2.726 | 2.222 | 6.354 |
|  | XRCC1 | n.p. | n.p. | 1.554 | 4.052 |
|  | FANCI | n.p. | n.p. | 2.897 | 26.101 |

*Tabelle S4: Selection of genes involved in DNA damage repair, cell cycle regulation and apoptosis analyzed in quiescent CD34^+^ treated with etoposide (1.5µM, and 10µM). Fold changes in mRNA expression were analyzed using qPCR and calculated with the 2-ΔΔCT method relative to untreated CD34^+^ cells and normalized to the reference gene RPL13A. The value 1 corresponds to a basic expression as in untreated cells; Values above 2 are considered a significant upregulation (green) while values below -2 are considered a significant downregulation (red) of a gene in treated CD34^+^ cells. Abbreviations: ETO, Etoposide; HR, Homologous Repair; NHEJ, Non-Homologous End Joining.*

|  | | **ETO 24h** | | |
| --- | --- | --- | --- | --- |
|  |  | 1.5µM ETO | 10µM ETO | |
| **Pathways** | **Gene** | **Fold Change** | |  |
| **Cell cycle regulation** | CDKN1A | 6.16 | 29.19 | |
|  | CDKN1B | -1.02 | -1.49 | |
|  | CCNB1 | -1.22 | -2.77 | |
|  | CCND1 | 1.18 | 1.16 | |
| **Apoptosis** | BAX | 1.63 | 1.64 | |
|  | BCL-2 | -1.14 | -1.41 | |
|  | FAS | 2.32 | 3.82 | |
| **NHEJ** | XRCC4 | -1.09 | -1.36 | |
| **HR** | RAD51 | -1.18 | -1.27 | |
| **Involved in DDR** | TP53 | 1.01 | -1.14 | |

Table S5: Selection of genes involved in DNA damage repair, cell cycle regulation and apoptosis analyzed in quiescent CD34^+^ treated with MNU (1mM, 3mM, and 5mM) for 1h. Fold changes in mRNA expression were analyzed using qPCR and calculated with the 2-ΔΔCT method relative to untreated CD34^+^ cells and normalized to the reference gene RPL13A. The value 1 corresponds to a basic expression as in untreated cells; Values above 2 are considered a significant upregulation (green) while values below -2 are considered a significant downregulation (red) of a gene in treated CD34^+^ cells. Abbreviations: HR, Homologous Repair; MNU, N-methyl-N-nitroso-urea; NHEJ, Non-Homologous End Joining; n.p., not performed.

|  | | **MNU d1** | | **MNU d3** | |
| --- | --- | --- | --- | --- | --- |
|  |  | 1mM MNU | 3mM MNU | 1mM MNU | 3mM MNU |
| **Pathways** | **Gene** | **Fold Change** | | | |
| **Direct repair** | MGMT | -1.877 | -1.841 | -1.170 | 1.249 |
| **Cell cycle regulation** | CDKN1A | 7.707 | 6.219 | 1.142 | 5.943 |
|  | CDKN1B | -1.720 | -1.506 | -293.575 | -265.398 |
|  | CCNB1 | -2.126 | -1.569 | -4.108 | -4.772 |
|  | CCND1 | -2.602 | -3.058 | -1.234 | 1.500 |
| **Apoptosis** | BAX | -1.301 | -1.034 | -886.362 | -371.693 |
|  | BCL-2 | -1.914 | -2.004 | -1.707 | 1.789 |
|  | CAS3 | -2.054 | -1.370 | -1.136 | 2.304 |
|  | CAS9 | -1.351 | 1.060 | 1.059 | 3.759 |
|  | CAS8 | -1.436 | -1.104 | -1.350 | -1.714 |
|  | FAS | 1.633 | 4.628 | 1.630 | 3.683 |
|  | BID | 1.102 | 1.667 | n.p. | n.p. |
| **NHEJ** | XRCC6 | -1.304 | -1.365 | -1.363 | 2.774 |
|  | XRCC4 | -1.786 | -1.951 | -307.726 | -230.717 |
|  | LIG4 | -1.217 | 1.242 | 1.497 | 4.897 |
|  | NHEJ1 | -1.412 | -128.783 | 1.135 | 1.935 |
| **HR** | RAD51 | -1.337 | -1.259 | -1.058 | 1.555 |
|  | BRCA1 | -4.492 | -6.207 | 1.131 | 2.377 |
|  | BRCA2 | -1.265 | -1.452 | 1.064 | 2.112 |
| **Autophagy** | ATG3 | -1.429 | -1.460 | -1.312 | 2.190 |
|  | BECN1 | -1.220 | -1.096 | 1.033 | 2.508 |
| **Quiescence** | mKi67 | n.p. | n.p. | 1.356 | 2.882 |
|  | CDK6 | n.p. | n.p. | 1.247 | 3.462 |
| **Involved in DDR** | H2AX | -1.996 | -2.295 | n.p. | n.p. |
|  | TP53 | -1.435 | -1.406 | 1.221 | 1.960 |
|  | APEX1 | -1.684 | -2.218 | -1.252 | 1.665 |
|  | FANCA2 | -1.140 | 1.074 | n.p. | n.p. |
